# An Enhanced Retroviral Vector for Efficient Genetic Manipulation and Selection in Mammalian Cells

**DOI:** 10.3390/biom14091131

**Published:** 2024-09-06

**Authors:** Jana Triller, Iryna Prots, Hans-Martin Jäck, Jürgen Wittmann

**Affiliations:** 1 Division of Molecular Immunology, Department of Internal Medicine III, Nikolaus-Fiebiger-Center of Molecular Medicine (NFZ), Friedrich-Alexander-Universität Erlangen-Nürnberg, Glückstraße 6, D-91054 Erlangen, Germany; 2 Department of Operative Dentistry and Periodontology, Friedrich-Alexander-Universität Erlangen-Nürnberg, Glückstraße 11, D-91054 Erlangen, Germany

**Keywords:** retrovirus, infection, B cells, FACS, EGFP, Puromycin, fusion protein, T2A, linker

## Abstract

Introducing genetic material into hard-to-transfect mammalian cell lines and primary cells is often best achieved through retroviral infection. An ideal retroviral vector should offer a compact, selectable, and screenable marker while maximizing transgene delivery capacity. However, a previously published retroviral vector featuring an EGFP/Puromycin fusion protein failed to meet these criteria in our experiments. We encountered issues such as low infection efficiency, weak EGFP fluorescence, and selection against infected cells. To address these shortcomings, we developed a novel retroviral vector based on the Moloney murine leukemia virus. This vector includes a compact bifunctional EGFP and Puromycin resistance cassette connected by a 2A peptide. Our extensively tested vector demonstrated superior EGFP expression, efficient Puromycin selection, and no growth penalty in infected cells compared with the earlier design. These benefits were consistent across multiple mammalian cell types, underscoring the versatility of our vector. In summary, our enhanced retroviral vector offers a robust solution for efficient infection, reliable detection, and effective selection in mammalian cells. Its improved performance and compact design make it an ideal choice for a wide range of applications involving precise genetic manipulation and characterization in cell-based studies.

## 1. Introduction

Retroviral infection is often the method of choice for efficiently introducing genetic information into difficult-to-transfect mammalian cell lines or primary cells [1,2]. In addition to lentiviral systems with increased safety requirements, retroviral systems based on the Moloney murine leukemia virus have proven to be effective in infecting primary hematopoietic cells in immunological research. In addition to cDNA delivery, infection can also be used to label cells with fluorescent proteins such as EGFP, allowing them to be identified by fluorescence microscopy or sorted by fluorescence-activated cell sorting (FACS). However, when working with large numbers of marker-positive cells, it is often desirable to pre-decimate non-infected cells by an effective selection step. In this context, Puromycin N-acetyltransferase (PAC) is often used, which renders infected cells insensitive to the naturally occurring aminonucleoside antibiotic Puromycin [3]. Cells are rapidly killed by Puromycin by inhibiting protein synthesis through ribosome-catalyzed incorporation into the C-terminus of elongating nascent chains. Blocking further elongation results in premature termination of translation and rapid cell death. Other advantages are the compact size of the PAC gene and the relatively low cost of Puromycin.

A common goal in retroviral vector design is to use as little space as possible for genetic elements due to limited coding capacity [4]. One way to achieve this is to link open reading frames (ORFs) using one or more internal ribosome entry site (IRES) elements [5], or to generate chimeric proteins that often combine screenable and selectable markers in a single marker protein. This can be accomplished either by direct fusion via a flexible amino acid (aa) linker [6] or by 2A “self-cleaving” peptides placed between ORFs that can induce ribosomal skipping during cellular protein translation [7]. Another important caveat to consider when designing retroviral vectors is the use of multiple promoters to express two or more genes, which can lead to transcriptional interference or promoter suppression [8].

A published retroviral vector [9] with an EGFP/Puromycin expression cassette was initially used to overexpress (c)DNA fragments and to both optically track and select for infected murine pro-B cells and B cell lines. However, our studies have shown that alterations at the C-terminus of the EGFP/Puro fusion protein can have a very detrimental effect on the functionality/stability of the entire fusion protein. In addition, infected and Puromycin-selected cells showed selection against the EGFP/Puro fusion protein. Therefore, we designed and tested an improved retroviral vector containing a bifunctional EGFP- and Puromycin resistance cassette linked by a 2A peptide. The functionality of the improved retroviral vector was directly compared with the originally used published vector. The enhanced vector demonstrated strong EGFP expression, efficient Puromycin selection, and no adverse effects on infected cells in various mammalian cell types, making this compact expression cassette the preferred choice for retroviral experiments requiring efficient infection, detection, and selection.

## 2. Materials and Methods

### 2.1. Cloning of Retroviral Vectors

#### 2.1.1. Cloning of pBMN-I-TagBFP

TagBFP cDNA was PCR amplified from the plasmid pMSCV-LTR-dCas9-VP64-BFP (Addgene plasmid # 46912) using the primers TagBFP NcoI for (GATCCATGGAGAGCGAGCTGATTAAGGAGAAC) and TagBFP SalI rev (GATGTCGACCACTGTGCTGGCGCTCTAATTAAGCTTGTGCCCCAGTTTGC) and Invitrogen™ Accu-Prime™ Pfx DNA-Polymerase (Thermo Fisher Scientific, Dreieich, Germany). All primers mentioned in the Materials and Methods section were ordered from Thermo Fisher Scientific and are shown in 5′ to 3′ orientation. The 734 bp PCR product was excised from a 1.0% agarose gel, purified with the QIAGEN gel extraction kit (QIAGEN, Hilden, Germany), and eluted in buffer EB. The PCR product was then digested with NcoI-HF and SalI-HF enzymes (NEB, Frankfurt, Germany) and ligated into the equally digested pBMN-I-GFP plasmid (Addgene plasmid # 1736). After electroporation of 1:20 volume of the ligation reaction into *E. coli* GeneHogs bacteria (Thermo Fisher Scientific, discontinued), cells were selected on LB/Amp plates at 37 °C overnight, single colonies were expanded, and isolated plasmid DNA was analyzed by restriction digests. PCR-amplified parts of clones with the correct digestion pattern were sequenced (Macrogen Europe BV, Amsterdam, The Netherlands) using primers EMCV IRES for (TCAACAAGGGGCTGAAGGAT) and MuLV 3 LTR seq rev (CCCCCCTTTTTCTGGAGACTAAAT). Correct clones were retransformed into heat shock-competent *E. coli* Stbl3 bacteria (Thermo Fisher Scientific). The complete plasmid sequence was confirmed using Oxford Nanopore Technologies (ONT) sequencing technology (Microsynth Seqlab GmbH, Göttingen, Germany) and designated pBMN-I-TagBFP. For transfection into human Platinum-E cells, all plasmid DNAs were isolated using the QIAGEN Plasmid Maxi Kit (QIAGEN).

#### 2.1.2. Cloning of pBMN-I-EGFP/Puro

The EGFP—linker—Puromycin (“EGFP/Puro”) cassette was generated as described for pEGFP-puro (Addgene plasmid # 45561; [9]). Briefly, the EGFP/Puro cassette was assembled by an overlap PCR reaction in which two PCR products with overlapping complementary sequences (underlined in the respective primers below) were generated and assembled in a subsequent overlap PCR reaction. In the first PCR reaction, EGFP was amplified from plasmid pCDH-EF1-MCS-BGH-PGK-EGFP-T2A-Puro (Wittmann lab, unpublished; details available upon request) using primers EGFP NcoI for (GATCCATGGTGAGCAAGGGCGAGGAG) and EGFP Puro linker rev (AAGGTCGTCTCCTAGATCTGAGTCCGGACTTGTACAGCTCGTCCATGC) and the AccuPrime™ Pfx DNA-Polymerase, yielding a 750 bp PCR product. In the second PCR reaction, the Puromycin resistance cassette from plasmid pCDH-EF1-MCS-BGH-PGK-EGFP-T2A-Puro was amplified using the primers EGFP Puro linker for (ACTCAGATCTAGGAGACGACCTTCCATGACCGAGTACAAGCCCACGGTG) and Puro SalI rev (GATGTCGACTCAGGCACCGGGCTTGCGGGTC) and the Accu-Prime™ Pfx DNA-Polymerase, resulting in a 634 bp PCR product. PCR products were excised from a 1.0% agarose gel, purified using the QIAGEN gel extraction kit, and eluted in buffer EB. For the overlap PCR reaction using the AccuPrime™ Pfx DNA-Polymerase, a 1:10 volume of each purified PCR product was used for initial PCR product annealing and elongation for 8 cycles. Outer primers EGFP NcoI for and Puro SalI rev were then added, and the reaction continued for another 22 cycles. The final 1361 bp overlap PCR product was excised from a 1.0% agarose gel, purified using the QIAGEN gel extraction kit, and eluted in buffer EB. The PCR product was subsequently digested with NcoI-HF and SalI-HF enzymes and ligated into the equally digested pBMN-I-GFP plasmid. After electroporation of a 1:20 volume of the ligation reaction into *E. coli* GeneHogs bacteria, cells were selected on LB/Amp plates at 37 °C overnight, single colonies were grown in liquid LB/Amp medium, and isolated plasmid DNA was analyzed by restriction digests. PCR-amplified parts of clones with the correct digestion pattern were sequenced using the primers EMCV IRES for EGFP rev (AAAGACCCCAACGAGAAGC) and MuLV 3 LTR seq rev. Correct clones were retransformed into heat shock-competent *E. coli* Stbl3 bacteria. The complete plasmid sequence was confirmed using ONT sequencing technology and designated pBMN-I-EGFP/Puro.

#### 2.1.3. Cloning of pBMN-I-EGFP/Puro Long

The C-terminus of PAC in pBMN-I-EGFP/Puro was modified by Gibson assembly. Briefly, pBMN-I-EGFP/Puro was digested with SalI-HF, treated with Calf intestinal phosphatase (NEB), and used together with the oligonucleotide PuroR long GA (CCTGGTGCATGACCCGCAAGCCCGGTGCGGGATCCACCGGATCTAGATAAGTCGACGATAAAATAAAAGATTTTATTTAG) in a Gibson assembly reaction using the “Gibson Assembly Master Mix” (NEB). After electroporation of a 1:30 volume of the Gibson assembly reaction into *E. coli* GeneHogs bacteria, cells were selected on LB/Amp plates at 37 °C overnight, single colonies were grown in liquid LB/Amp medium, and isolated plasmid DNA was analyzed by restriction digests. The region containing the inserted oligo from clones with the correct digestion pattern was sequenced using the primer Puromycin for (CTCGACATCGGCAAGGTGTG). Correct clones were retransformed into heat shock-competent *E. coli* Stbl3 bacteria. The complete plasmid sequence was confirmed using ONT sequencing technology and designated pBMN-I-EGFP/Puro long.

#### 2.1.4. Cloning of pBMN-I-EGFP/Puro Myc Long

A Myc tag was added to the C-terminus of PAC in pBMN-I-EGFP/Puro long by Gibson assembly. Briefly, pBMN-I-EGFP/Puro long was digested with SalI-HF, treated with Calf intestinal phosphatase, and used together with the oligonucleotide PuroR Myc long GA (GCCCGGTGCGGGATCCACCGGATCTAGAGAGCAGAAACTCATCTCAGAAGAGGATCTGTAAGTCGACGATAAAATAAAAGATTTTATTT) in a Gibson assembly reaction using the “Gibson Assembly Master Mix”. After electroporation of a 1:30 volume of the Gibson assembly reaction into *E. coli* GeneHogs bacteria, cells were selected on LB/Amp plates at 37 °C overnight, single colonies were grown in liquid LB/Amp medium, and isolated plasmid DNA was analyzed by restriction digests. The region containing the inserted oligo from clones with the correct digestion pattern was sequenced using the primer Puromycin for. Correct clones were retransformed into heat shock-competent *E. coli* Stbl3 bacteria. The complete plasmid sequence was confirmed using ONT sequencing technology and designated pBMN-I-EGFP/Puro Myc long.

#### 2.1.5. Cloning of pBMN-I-EGFP-T2A-Puro

The EGFP-T2A-Puro cassette was PCR amplified from plasmid pCDH-EF1-MCS-BGH-PGK-EGFP-T2A-Puro using primers EGFP NcoI for and Puro SalI rev and the AccuPrime™ Pfx DNA-Polymerase. The 1385 bp PCR product was excised from a 1.0% agarose gel, purified with the QIAGEN gel extraction kit, and eluted in buffer EB. The PCR product was then digested with NcoI-HF and SalI-HF enzymes and ligated into the equally digested pBMN-I-GFP plasmid. After electroporation of a 1:20 volume of the ligation reaction into *E. coli* GeneHogs bacteria, cells were selected on LB/Amp plates at 37 °C overnight, single colonies were grown in liquid LB/Amp medium, and isolated plasmid DNA was analyzed by restriction digests. Clones with the correct digestion pattern were sequenced using primers EMCV IRES for EGFP rev and MuLV 3 LTR seq rev. Correct clones were retransformed into heat shock-competent *E. coli* Stbl3 bacteria. The complete plasmid sequence was confirmed using ONT sequencing technology and designated pBMN-I-EGFP-T2A-Puro.

#### 2.1.6. Cloning of pBMN-I-EGFP-T2A-Puro Myc

A Myc tag was added to the C-terminus of PAC in pBMN-I-EGFP-T2A-Puro by Gibson assembly. Briefly, pBMN-I-EGFP-T2A-Puro was digested with SalI-HF, treated with Calf intestinal phosphatase, and used together with the oligonucleotide PuroR Myc GA (CTGGTGCATGACCCGCAAGCCCGGTGCCGAGCAGAAACTCATCTCAGAAGAGGATCTGTAAGTCGACGATAAAATAAAAGATTTTATTT) in a Gibson assembly reaction using the “Gibson Assembly Master Mix”. After electroporation of a 1:30 volume of the Gibson assembly reaction into *E. coli* GeneHogs bacteria, cells were selected on LB/Amp plates at 37 °C overnight, single colonies were grown in liquid LB/Amp medium, and isolated plasmid DNA was analyzed by restriction digests. The region containing the inserted oligo from clones with the correct digestion pattern was sequenced using the primer Puromycin for. Correct clones were retransformed into heat shock-competent *E. coli* Stbl3 bacteria. The complete plasmid sequence was confirmed using ONT sequencing technology and designated pBMN-I-EGFP-T2A-Puro Myc.

### 2.2. Cell Lines and Culture Conditions

Murine 38B9 pro-B cells [10] were cultured in RPMI 1640 medium supplemented with 10% fetal bovine serum, 1% L-glutamine, 1% sodium pyruvate, 1% penicillin/streptomycin, and 0.1% β-mercaptoethanol (all from Thermo Fisher Scientific) at 37 °C and 5% CO_2_ in a humidified incubator. The murine fibroblast cell line NIH3T3 (ATCC cat. No. CRL-1658; [11]) and the ecotropic human packaging cell line Platinum-E (Cell Biolabs, Inc., San Diego, CA, USA; [12]) were grown in DMEM medium supplemented with 10% fetal bovine serum, 1% L-glutamine, and 1% penicillin/streptomycin (all from Thermo Fisher Scientific) and maintained at 37 °C and 7.5% CO_2_ in a humidified incubator.

### 2.3. Transient Transfection of Platinum-E Cells for Retrovirus Production

24 h before transfection, 3.5 × 10^6^ Platinum-E cells were plated in 10 mL complete DMEM medium in 100 mm dishes (Greiner Bio-One, Frickenhausen, Germany). For transient transfection on the next day, an Eppendorf cup was prepared with 15 µg of retroviral expression vector diluted in a total volume of 222 µL OptiMEM medium without supplements (Thermo Fisher Scientific) and 72 µL PEI (Polyethylenimine “Max”, (Mw 40,000)—High Potency Linear PEI, 1 mg/mL; Polysciences Inc., Warrington, PA, USA) was added. After brief vortexing, the mixture was incubated at room temperature for 10 min and added dropwise to Platinum-E cells. After 5 h of incubation at 37 °C, the DNA/PEI/DMEM medium was carefully removed and replaced with 10 mL complete DMEM medium. The supernatant was collected 72 h after transfection, filtered through 0.45 µm syringe filters (Sartorius, Göttingen, Germany), and either used directly for retroviral infection or stored at −80 °C until use. To determine the transfection efficiency, Platinum-E cells were carefully washed with PBS after removal of the retroviral supernatant, trypsinized, and analyzed for EGFP- and TagBFP fluorescence by flow cytometry.

### 2.4. Retroviral Infection of 38B9- and NIH3T3 Cells

Retroviral infection was performed by spinoculation [13]. Briefly, 2.5 × 10^4^ NIH3T3 cells were seeded in 1 mL complete DMEM medium in a 24-well plate (Greiner Bio-One) one day before infection. On the day of infection, the medium was removed and replaced with 1 mL retroviral culture supernatant from Platinum-E cells transfected with the respective vectors plus 4 µg/mL polybrene (Sigma-Aldrich, Taufkirchen, Germany). For 38B9 suspension cells, cells were counted and 1 × 10^6^ cells harvested by centrifugation. Cell pellets were resuspended in 1 mL retroviral culture supernatant containing 4 µg/mL polybrene and transferred to a 24-well plate. Both cell lines were centrifuged at 1700× *g* for 3.5 h at 33 °C to achieve close contact between the retroviral particles and the cells. Retroviral supernatants were then removed, and cell culture was continued in fresh medium under the appropriate conditions. Live cells were analyzed for EGFP- and TagBFP expression by flow cytometry on the day of infection and every day thereafter for up to 5 days. Results presented are from one of three independent experiments.

### 2.5. Structural Alignment of Predicted EGFP/Puro Fusion Proteins

The structure of the EGFP/Puro fusion proteins was predicted using AlphaFold [14]. A Colab notebook for AlphaFold was used (https://colab.research.google.com/github/deepmind/alphafold/blob/main/notebooks/AlphaFold.ipynb, accessed on 15 July 2024) and queried with the EGFP/Puro protein sequences of both pBMN-I-EGFP/Puro and pBMN-I-EGFP/Puro long. The Protein Data Bank’s “Pairwise Structure Alignment” tool [15] was used to assess structural similarity, including the calculation of the template modeling (TM) score [16] and the root mean square deviation (RMSD) value. All generated PDB files are available as supplemental materials.

### 2.6. Western Blot Analysis

Retrovirally infected and subsequently 5 days Puromycin-selected 38B9- or NIH3T3 cells as well as wild-type cells were washed twice in PBS buffer, and the cell pellet was resuspended in 2× Laemmli loading buffer (4% SDS, 20% Glycerol, 125 mM Tris, pH 6.8, 10% β-mercaptoethanol, 0.002% Bromphenolblue; [17]) at 33 µL per 1 × 10^6^ cells. The suspension was pipetted through a syringe with a 23G needle (BD Microlance 3; BD, Heidelberg, Germany) 4 times to shear genomic DNA, boiled at 100 °C for 6 min, and stored on ice. For SDS-PAGE molecular weight estimation, the prestained PageRuler protein ladder (Thermo Fisher Scientific) was used. Lysates corresponding to 1 × 10^6^ cells were separated on 13.5% SDS-PAA gels, transferred to 0.2 µm nitrocellulose membranes (Amersham Protran, Cytiva Europe GmbH, Freiburg, Germany), and subjected to Western blot analysis as previously described [18]. Briefly, the membranes were blocked with 5% nonfat dry milk (Carl Roth GmbH + Co. KG, Karlsruhe, Germany) in Tris-buffered saline with Tween 20 (TBST), incubated with the appropriate unconjugated primary antibody followed by suitable secondary HRP-conjugated antibodies (Bio-Rad Laboratories GmbH, Feldkirchen, Germany), and developed with the Enhanced Chemiluminescence (ECL) method using the Trident femto Western HRP Substrate (GeneTex Inc., Irvine, CA, USA). Signals using monoclonal mouse anti-GFP (Clones 7.1 and 13.1, Roche, distributed by Merck, Darmstadt, Germany), monoclonal mouse anti-c-Myc (clone 9E10; [19]), and polyclonal rabbit anti-β-actin antibodies (Sigma-Aldrich, cat. No. A2066) were visualized as described above. Results presented are from one of two independent experiments.

### 2.7. Flow Cytometry

Trypsinized infected NIH3T3 cells and infected 38B9 suspension cells were analyzed using a Gallios flow cytometer (Beckman, Krefeld, Germany). Scattered light was measured by the forward- (FS INT) and sideward (SS INT) scatter detectors to identify viable cells. A plot of the FS peak signal (FS PEAK) against the area-based FS intensity (FS INT) allowed the exclusion of multimeric cell agglomerates. EGFP fluorescence intensity was measured in the FL-1 channel, and TagBFP fluorescence intensity was measured in the FL-9 channel. Raw data were analyzed using Kaluza Analysis software version 2.1 (Beckman).

## 3. Results

The method of choice for introducing transgenes into suspension cells of the murine hematopoietic system is retroviral transduction. The most efficient system is based on the ecotropic Moloney murine leukemia virus and derived pBMN vectors. A commonly used retroviral construct is the plasmid pBMN-I-GFP, which carries an EGFP fluorescent marker and a multiple cloning site for transgene insertion (Supplemental Figure S1). Since the infection efficiency of primary hematopoietic cells is usually not very high and not every laboratory has access to a FACS to sort large numbers of infected fluorescent cells, an antibiotic resistance conferring expression cassette for selection would be very helpful when transducing large numbers of cells. Since this is not possible with pBMN-I-GFP, a cassette as in the published pBMN-based bifunctional retroviral vector pMA73 would be desirable [9]. This vector contains the EGFP ORF fused to the PAC ORF by ten linker-encoded aa. With this compact marker cassette, it should be possible to both optically track and select infected cells while still leaving enough coding capacity to introduce even large genetic elements. Since pMA73 was not available from Addgene, we decided to construct the EGFP/Puro cassette ourselves and insert it into the pBMN vector. Looking at the Materials and Methods section of the corresponding manuscript [9], we noticed some discrepancies. For example, the PAC ORF is reported there to be 200 aa [when in fact it is 199 aa, which is also consistent with the available sequence provided by this group at https://www.addgene.org/45561/ (accessed on 15 July 2024)], or the PAC C-terminus is reported in the manuscript to be extended by 11 aa residues due to the subcloning strategy, when in fact it is only six aa according to the available sequence. To be on the safe side, we decided to use the canonical PAC ORF sequence reported in UniProt [https://www.uniprot.org/uniprotkb/P13249/entry (accessed on 15 July 2024)]. The EGFP- and PAC ORFs were joined in an overlap PCR reaction as described in the Materials and Methods section of [9], sequenced, and cloned into pBMN-I-GFP (which is named “GFP” but actually contains the “EGFP” fluorescent protein ORF), yielding pBMN-I-EGFP/Puro (Supplemental Figure S2). In parallel, another pBMN-I-GFP variant was created carrying the ORF of the blue fluorescent protein TagBFP instead of EGFP. This retroviral vector pBMN-I-TagBFP (Supplemental Figure S3) can be used, for example, for gating in flow cytometric analyses since the cells are infected, but its blue fluorescence does not interfere with EGFP detection in flow cytometric analyses.

**Figure 1:**
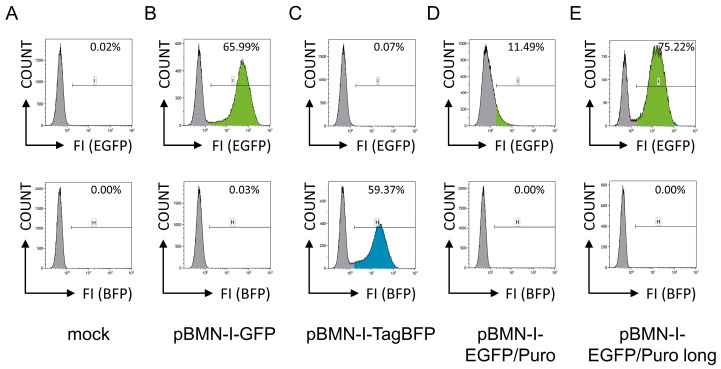
Flow cytometric analyses for EGFP- and TagBFP fluorescence of 38B9 cells infected with different retroviral supernatants. 38B9 cells were (**A**) mock infected or infected with different retroviral supernatants derived from (**B**) pBMN-I-GFP, (**C**) pBMN-I-TagBFP, (**D**) pBMN-I-EGFP/Puro, and (**E**) pBMN-I-EGFP/Puro long and incubated for one day before flow cytometric analysis to determine infection efficiencies. A detailed gating and analysis strategy is shown in Supplemental Figure S4. Cells (COUNT) are shown in histogram plots for EGFP- [FI (EGFP), gate “I”, upper row] or BFP- [FI (BFP), gate “H”, bottom row] fluorescence intensities. Percentages of gated cells are shown in the graphs.

In a pilot experiment, the murine pro-B cell line 38B9 [10] was infected with retroviral supernatants generated with these constructs. The packaging cell line Platinum-E, which carries all necessary proteins for the assembly of retroviral particles [12], was transiently mock transfected or transfected with pBMN-I-GFP, pBMN-I-TagBFP, and pBMN-I-EGFP/Puro constructs, and retroviral particle-containing supernatants were harvested 72 h later. For retroviral infection of 38B9 cells by spinoculation, equal amounts of supernatant were used, and cells were analyzed for EGFP- and TagBFP fluorescence by flow cytometric analyses one day after infection. 38B9 cells infected with supernatant from mock-transfected Platinum-E cells were used to adjust optimal FACS settings (Figure 1A). The detailed flow cytometric gating strategy as well as representative flow cytometric analyses for EGFP- and TagBFP fluorescences of infected 38B9 cells are shown in Supplemental Figure S4. While cells infected with pBMN-I-GFP (Figure 1B) and pBMN-I-TagBFP (Figure 1C) showed infection efficiencies of approximately 66% and 59%, respectively, pBMN-I-EGFP/Puro-infected cells showed an infection efficiency of only approximately 11% (Figure 1D). In addition, the EGFP fluorescence intensity of these cells was significantly lower and no distinct infected cell population was observed. These findings were reproducible in three independent experiments using retroviral supernatants generated from independent plasmid preparations, fully sequence-verified via Oxford Nanopore technology.

In addition, the same results were observed in a second cell line, the murine adherent cell line NIH3T3 (Supplemental Figure S5; [11]). This may indicate an inherent problem with the EGFP/Puro cassette in infections with pBMN-I-EGFP/Puro-derived retroviral particles.

### 3.1. Modifying the EGFP/Puro Cassette by Extending the PAC C-Terminus

The results of the FACS analyses were puzzling, as we would have expected the EGFP fluorescence intensity of the published EGFP/Puro cassette of pBMN-I-EGFP/Puro to be comparable to that of pBMN-I-GFP. The only reason we could think of is the shortening of the EGFP/Puro fusion protein by six aa compared with the originally described vector. To see if this actually has an effect, we modified the PAC C-terminus and added the six aa (“GSTGSR”) present in the available sequence information of the EGFP/Puro cassette, resulting in pBMN-I-EGFP/Puro long (Supplemental Figure S6). Again, the murine pro-B cell line 38B9 was infected with retroviral supernatants generated with this adapted construct and analyzed for EGFP fluorescence by FACS analysis. Surprisingly, the number of EGFP-positive cells increased significantly compared with both pBMN-I-EGFP/Puro and pBMN-I-GFP (Figure 1E). While the intensity of EGFP also increased in pBMN-I-EGFP/Puro long-infected cells relative to pBMN-I-EGFP/Puro, it did not quite reach the levels observed with pBMN-I-GFP. This was also true when NIH3T3 cells were infected (Supplemental Figure S5E).

**Figure 2:**
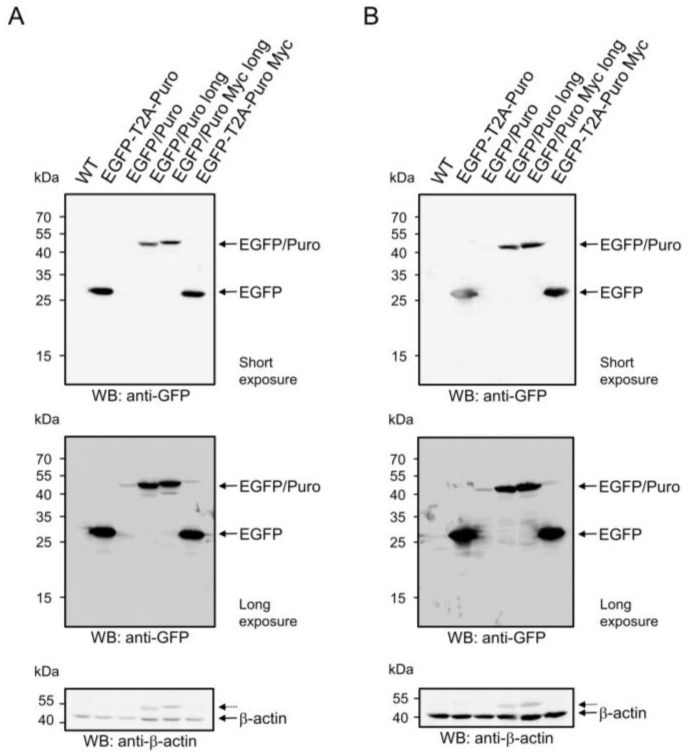
Western blot analysis of EGFP proteins in wild-type- and infected 38B9- and NIH3T3 cells. Cell lysates from 1 × 10^6^ uninfected and unselected (**A**) 38B9- or (**B**) NIH3T3 cells (“WT”) or from 1 × 10^6^ (**A**) 38B9- or (**B**) NIH3T3 cells infected with retroviral supernatant from the respective vectors pBMN-I-EGFP-T2A-Puro (“EGFP-T2A-Puro”), pBMN-I-EGFP/Puro (“EGFP/Puro”), pBMN-I-EGFP/Puro long (“EGFP/Puro long”), pBMN-I-EGFP/Puro Myc long (“EGFP/Puro Myc long”) or pBMN-I-EGFP-T2A-Puro Myc (“EGFP-T2A-Puro Myc”) and Puromycin-selected for 5 days were reduced, separated by 13.5% SDS-PAGE, and transferred to a nitrocellulose membrane. The membrane was blocked, stained with monoclonal mouse anti-GFP antibodies, and developed with an appropriate HRP-conjugated secondary antibody using the ECL method for a short (upper panel) or prolonged exposure (middle panel). The loading of same cell equivalents was assessed with polyclonal rabbit antibodies against beta-actin (bottom panel). Dashed arrows in the beta-actin blots indicate signals from previous probing with the GFP antibody. Original images can be found in Supplemental Figures S9 and S10.

To investigate whether the observed differences in EGFP fluorescence intensities between pBMN-I-EGFP/Puro and pBMN-I-EGFP/Puro long could be due to differences in the amount of EGFP/Puro fusion protein expressed after infection, Puromycin-selected 38B9 cells infected with supernatants from the respective vectors and 38B9 WT cells were subjected to Western blot analysis for EGFP amounts. As an additional control, a modified version of pBMN-I-EGFP/Puro long with a C-terminal Myc tag named pBMN-I-EGFP/Puro Myc long was included (Supplemental Figure S7). While the EGFP/Puro fusion proteins in lysates of 38B9 cells infected with pBMN-I-EGFP/Puro long and pBMN-I-EGFP/Puro Myc long showed clear signals at calculated molecular masses of 50.1 kDa and 51.3 kDa, respectively, lysates of 38B9 cells infected with pBMN-I-EGFP/Puro (calculated molecular mass 49.6 kDa) showed a weak signal for the EGFP/Puro fusion protein only after overexposure (Figure 2A, middle panel). Wild-type 38B9 cells showed no signal for EGFP in Western blot analysis. The same results were observed in NIH3T3 cells (Figure 2B, upper and middle panels). Western blot analysis of β-actin levels excluded loading of unequal amounts of protein as a reason for the observed differences in EGFP/Puro protein abundance (Figure 2, bottom panels).

To rule out the trivial possibility that the consistently observed differences in EGFP fluorescence intensity and EGFP/Puro fusion protein abundance were due to differences in the number of viral particles used for infection, the relative retroviral titers were quantified by measuring reverse transcriptase activity in the retroviral supernatants using the SG-PERT assay ([20]; see Appendix A.1 in Appendix A, Supplementary Methods for further experimental details). Quantitative real-time PCR analyses of the retroviral supernatants of all vectors used in this study and tenfold dilutions thereof showed very comparable relative retroviral titers, excluding large differences in retroviral titers as a reason for the observed effect (Supplemental Figure S8) and suggesting that the changes at the PAC C-terminus may be related to the stability and/or function of the fusion protein.

To investigate whether the six aa added to the PAC C-terminus could possibly affect the structure of the fusion protein, we predicted both structures using AlphaFold [14]. A Colab notebook for AlphaFold was used and queried with the protein sequences of the EGFP/Puro fusion proteins from both pBMN-I-EGFP/Puro and pBMN-I-EGFP/Puro long. The “Pairwise Structure Alignment” tool of the Protein Data Bank [15] was used to assess structural similarity.

Surprisingly, the addition of the six aa at the PAC C-terminus resulted in a profound change in the prediction of both the EGFP and the PAC portions of the fusion protein (Supplemental Figure S11, Supplemental Materials). The template modeling (TM) score, which is a measure of the topological similarity between the two structures, was reduced to 0.82, where a value of 1 indicates a perfect match and 0 indicates no match between the two structures. Similarly, the root mean square deviation (RMSD) value of both fusion proteins is 3.77 Å. The lower the RMSD value, the better the structure alignment between the pair of structures. We conclude that the prediction results indicate that adding six aa to the PAC C-terminus may induce folding differences and profound structural changes in both fusion protein parts, possibly altering protein stability and/or function.

In conclusion, the addition of a few aa at the PAC C-terminus of EGFP/Puro fusion proteins is predicted to result in markedly altered structures, which could lead to profound differences in the amount and functionality of EGFP/Puro fusion proteins and thus EGFP fluorescence intensities in infected cells, despite infection with comparable numbers of retroviral particles.

### 3.2. Testing the Functionality of the Puromycin Resistance Cassette

**Figure 3:**
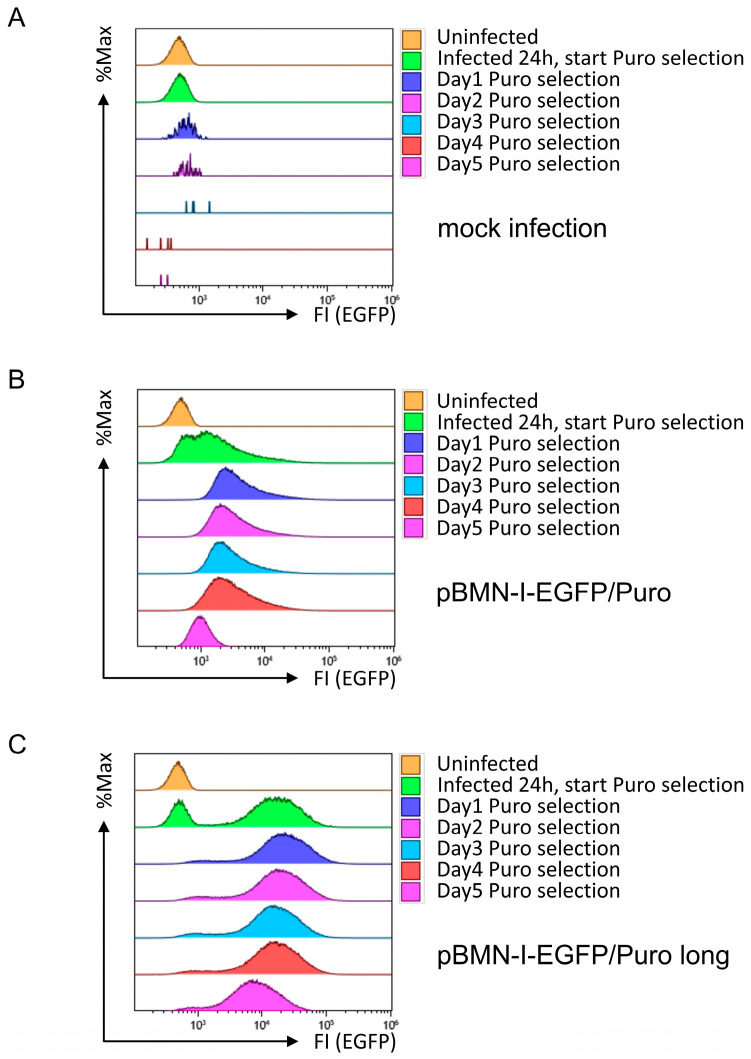
Flow cytometric analyses for EGFP fluorescence in 38B9 cells infected with EGFP/Puro cassette-containing retroviral supernatants and subsequent Puromycin selection. 38B9 cells were infected with retroviral supernatants derived from (**A**) mock-, (**B**) pBMN-I-EGFP/Puro-, or (**C**) pBMN-I-EGFP/Puro long-transfected Platinum-E cells. Puromycin was added at 5 µg/mL 24 h after infection. EGFP fluorescence intensities to determine infection efficiency were measured by flow cytometry on the day of infection, one day after infection, and every other day for 5 days. Data acquisition and gating strategy were identical to those described in Figure 1. EGFP fluorescence intensities of FSC/SSC-gated single cells are presented as overlay histograms, and relative cell numbers of each measurement are normalized and presented as %Max.

The second prerequisite for the retroviral vector was the ability to rapidly and efficiently eliminate uninfected cells by antibiotic selection. To test this requirement, Puromycin was first pre-titered on 38B9 cells, and an optimal concentration of 5 µg/mL Puromycin was determined (see Appendix A.2 in Appendix A, Supplementary Methods for further experimental details). Subsequently, 38B9 cells treated with control supernatant or infected with retroviral supernatants derived from pBMN-I-EGFP/Puro or pBMN-I-EGFP/Puro long, as shown in Figure 1, were treated with 5 µg/mL Puromycin one day after infection. Cells were then monitored for five consecutive days by flow cytometric analysis, assessing forward- and sideward scattering properties (as an approximation of cell viability) and EGFP fluorescence in single cells. 38B9 cells infected with supernatant from mock-transfected Platinum-E cells died very quickly after application of selection pressure (Figure 3A). 38B9 cells infected with pBMN-I-EGFP/Puro-generated supernatant showed both successful infection and selection, as EGFP-positive cells were detectable after infection and presumably uninfected EGFP-negative cells disappeared after applying Puromycin selection (Figure 3B). This is even more pronounced for 38B9 cells infected with pBMN-I-EGFP/Puro long-derived supernatant, which showed a peak in EGFP fluorescence intensity that was approximately 1 log shift stronger than cells infected with pBMN-I-EGFP/Puro (Figure 3C).

Interestingly, the low amounts of EGFP/Puro fusion protein in cells infected with pBMN-I-EGFP/Puro appear to be sufficient for efficient Puromycin selection, as the number of live 38B9 cells was reduced after 24 h of selection in both cases. Most strikingly, EGFP fluorescence intensity decreased in both infected cell lines over the course of 5 days of continued selection pressure. This result was reproducible and also observed in the Myc-tagged construct pBMN-I-EGFP/Puro Myc long (Supplemental Figure S12). Since this effect also occurred in other cell lines, such as NIH3T3 cells (Supplemental Figure S13), this finding suggests that there is a selection against cells expressing either the short or long EGFP/Puro expression cassette.

### 3.3. Cloning and Testing of an Improved Retroviral Vector with EGFP/Puro Cassette

Therefore, we decided to develop an alternative vector with a compact, selectable, and screenable marker. Instead of a ten-aa linker peptide, the sequence encoding a T2A “self-cleaving” peptide was used to link the EGFP- and PAC ORFs, and the resulting cassette was cloned into pBMN-I-GFP, resulting in plasmid pBMN-I-EGFP-T2A-Puro (Supplemental Figure S14). A C-terminally Myc-tagged version, pBMN-I-EGFP-T2A-Puro Myc (Supplemental Figure S15), was also generated to allow later assessment of Puromycin protein amounts. 38B9 cells were infected with supernatant generated from mock transfected Platinum-E cells or cells transfected with the new retroviral constructs. Infection efficiencies were approximately 68% or 60% as determined by flow cytometric analysis, with efficiencies at least as good as those achieved with pBMN-I-GFP (Supplemental Figure S16).

**Figure 4:**
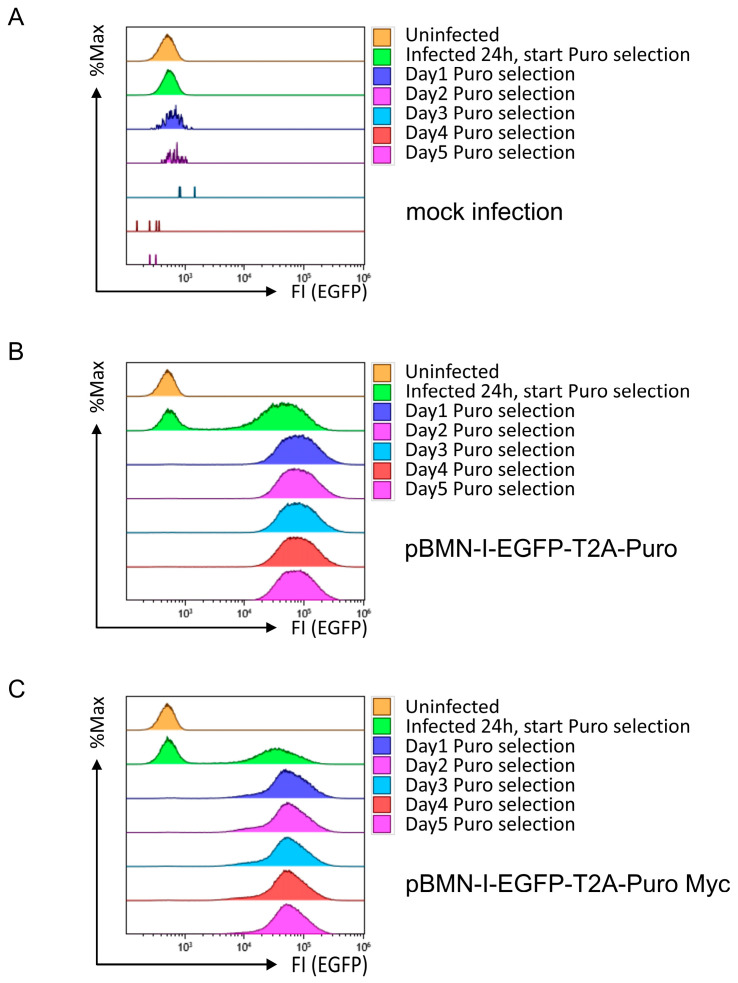
Flow cytometric analyses for EGFP fluorescence in 38B9 cells infected with EGFP-T2A-Puro cassette-containing retroviral supernatants and subsequent Puromycin selection. 38B9 cells were infected with retroviral supernatants derived from (**A**) mock-, (**B**) pBMN-I-EGFP-T2A-Puro-, or (**C**) pBMN-I-EGFP-T2A-Puro Myc-transfected Platinum-E cells. Puromycin was added at 5 µg/mL 24 h after infection. EGFP fluorescence intensities to determine infection efficiency were measured by flow cytometry on the day of infection, one day after infection, and every other day for 5 days. Data acquisition and gating strategy were identical to those described in Figure 1. Data of mock infection are identical to those in Figure 3. EGFP fluorescence intensities of FSC/SSC-gated single cells are presented as overlay histograms, and relative cell numbers of each measurement are normalized and presented as %Max.

To test the functionality of the Puromycin cassette in this new setup, the same infection and selection experiment as in Figure 3 was performed. Wild-type 38B9 cells treated with control supernatant died very quickly after application of selection pressure (Figure 4A), whereas 38B9 cells infected with retroviral supernatants derived from pBMN-I-EGFP-T2A-Puro or pBMN-I-EGFP-T2A-Puro Myc showed both successful infection and selection, as EGFP-positive cells were visible after infection and presumably uninfected EGFP-negative cells disappeared after application of Puromycin selection (Figure 4B,C). EGFP fluorescence intensity was almost 1 log shift stronger than with pBMN-I-EGFP/Puro long constructs and even stronger than with pBMN-I-GFP. More importantly, the EGFP fluorescence intensity remained constant over time, indicating that this modified EGFP/Puro cassette is superior to the old construct. These results were confirmed using NIH3T3 cells (Supplemental Figure S17).

The efficiency of self-cleavage of the EGFP-T2A-Puro fusion proteins was assessed by analysis of EGFP amounts. Puromycin-selected 38B9 cells infected with supernatants of the respective T2A-bearing vectors and wild-type 38B9 cells were subjected to Western blot analysis for EGFP amounts (Figure 2A, upper panel). Cells infected with retroviral particles of both T2A constructs, pBMN-I-EGFP-T2A-Puro and pBMN-I-EGFP-T2A-Puro Myc, clearly showed a signal at a calculated molecular mass for processed EGFP of 28.7 kDa, whereas wild-type 38B9 cells showed no EGFP signal in Western blot analysis. The EGFP signal intensities of the T2A-generated EGFP proteins were stronger than the signals of the EGFP/Puro fusion proteins. The results for 38B9 cells are consistent with those for NIH3T3 cells (Figure 2B, upper panel). The almost complete absence of full-length EGFP-T2A-Puro proteins (predicted molecular masses of 50.2 kDa and 51.4 kDa) indicates a very efficient self-cleavage of the fusion proteins in both 38B9- and NIH3T3 cells. Western blot analysis of β-actin levels excluded loading of unequal amounts of protein as a reason for the observed differences in EGFP protein abundance (Figure 2A,B, bottom panels).

To obtain an estimate of PAC amounts in infected and selected cells, in the absence of a functional PAC antibody, we used Myc-tagged variants as surrogates for Western blot analysis. Puromycin-selected 38B9 cells infected with supernatants of the respective Myc-tagged vectors and wild-type 38B9 cells were subjected to Western blot analysis. Cells infected with retroviral particles of pBMN-I-EGFP/Puro Myc long showed a strong signal at the calculated molecular mass of 51.3 kDa, whereas the T2A-processed PAC fragment showed a comparatively weak signal at the calculated molecular mass of 22.8 kDa. Wild-type 38B9 cells showed no Myc signal in Western blot analysis (Supplemental Figure S18A, upper panel). The same data were obtained for NIH3T3 cells (Supplemental Figure S19A, upper panel; original images can be found in Supplemental Figures S18B and S19B). Similar to the finding of efficient Puromycin selection with low amounts of EGFP/Puro fusion proteins, the T2A-produced PAC protein appears to be sufficient for efficient selection. Western blot analysis of β-actin levels excluded loading of unequal amounts of protein as a reason for the observed differences in Myc-tagged protein abundance (Supplemental Figures S18A and S19A, bottom panels).

In summary, retroviral infection with supernatants generated with the novel pBMN-I-EGFP-T2A-Puro vector results in higher numbers of infected cells and higher EGFP fluorescence intensities than supernatants generated with vectors containing the published EGFP/Puro cassette, most likely due to higher EGFP protein levels. This novel vector is at least as efficient as retroviral supernatants generated with the commonly used pBMN-I-GFP vector, which, however, does not allow selection. Importantly, there is no selection against infected cells, as is the case with the published EGFP/Puro cassette. All of these findings apply to both suspension and adherent cells. In conclusion, these data make the plasmid pBMN-I-EGFP-T2A-Puro the construct of choice for successful retroviral infection, selection, and visualization of mammalian suspension and adherent cells.

## 4. Discussion

To overcome the challenges and inefficiencies of transfecting murine B cells, we opted for retroviral transduction to achieve fast, efficient, and stable transgene expression. We sought a retroviral vector that would enable tracking and sorting of infected cells via FACS and allow for the rapid elimination of uninfected cells using Puromycin selection. Additionally, we required a vector with high transgene capacity. A suitable retroviral vector based on the Moloney murine leukemia virus, described in the literature [9], features a bifunctional EGFP/Puromycin expression cassette (pMA73) available from Addgene. For reconstructing this vector (referred to as pBMN-I-EGFP/Puro in our manuscript), we used the PAC C-terminus sequence from the UniProt database due to inconsistencies in the original description.

Repeated infection experiments with supernatants derived from transfections of a packaging cell line with this retroviral vector backbone showed, in contrast to supernatants from the “standard” retroviral vector pBMN-I-GFP, only very low infection efficiencies and low EGFP fluorescence intensities. This was true for 38B9 suspension cells and NIH3T3 adherent cells. Since the published pBMN-I-EGFP/Puro vector did not work as expected, we suspected that this might be due to the introduced changes at the PAC C-terminus. We added the six aa to the C-terminus of PAC, which were originally added during the subcloning procedure of this vector as described in [9]. Their addition did indeed restore EGFP fluorescence intensities in FACS analyses to levels similar to pBMN-I-GFP. Western blot analysis of infected cells using an EGFP antibody showed that the longer EGFP/Puro fusion protein was present in reasonable amounts, while the amount of the shorter fusion protein was drastically reduced, likely explaining the very low infection efficiencies and low EGFP fluorescence intensities. Structure predictions of both EGFP/Puro fusion proteins using AlphaFold suggest that the addition of six C-terminal aa leads to profound structural changes in both parts of the fusion protein, likely resulting in decreased protein stability or half-life. To exclude that the observed large differences between the retroviral constructs were due to lower particle numbers in the retroviral supernatant preparations derived from pBMN-I-EGFP/Puro, the reverse transcriptase activities of all retroviral supernatants used in this study were quantified. This assay is an accurate, rapid, and relatively inexpensive method with higher sensitivity than antigen or genome detection methods [20]. A dilution range of approximately five orders of magnitude could be used to assess reverse transcriptase activity, leading us to conclude that the titers of all retroviral supernatants were quite comparable. Unrecognized mutations introduced during the cloning procedures as a second possibility for the observed large differences between the retroviral constructs were excluded by complete sequencing of all plasmids used in this study by Oxford Nanopore sequencing.

To investigate the functionality of the Puromycin resistance cassettes, we selected 38B9 and NIH3T3 cells one day after infection with Puromycin for up to 5 days and subsequently analyzed infection efficiencies and EGFP fluorescence intensities by flow cytometry. Selection of cells infected with supernatants from retroviral vectors with both long and short PAC C-termini showed a rapid and efficient accumulation of almost exclusively EGFP-positive cells, indicating a very good functionality of the PAC expression cassette, even for the less abundant short EGFP/Puro fusion protein.

The observation that the number of cells infected with retroviral particles derived from all three vectors containing the EGFP/Puro selection cassette declined over time in all cell lines tested made it impossible for us to use the vector. We cannot rule out the possibility that the observed decline in infected cells is specific to the cell lines or conditions we tested, as we were unable to conduct experiments with the cells and conditions used by [9]. Since we do not currently know whether the fusion protein is harmful to the cells or whether the integrated vector is affected by factors such as transgene silencing [21], we decided to modify the EGFP/Puro cassette to avoid counter-selection of infected cells and potentially achieve higher infection efficiencies and EGFP fluorescence intensities. The linker peptide in the EGFP/Puro cassette was replaced by a “self-cleaving” T2A peptide, resulting in the creation of plasmid pBMN-I-EGFP-T2A-Puro. Infection experiments with this novel vector showed that it was at least as efficient as pBMN-I-GFP in infecting 38B9- and NIH3T3 cells. Cells infected with pBMN-I-EGFP-T2A-Puro showed a clearly separable population of EGFP-positive cells whose peak EGFP fluorescence intensity was approximately 2 log shifts stronger than that of the non-infected cell population, allowing efficient sorting by FACS. Western blot analysis of 38B9- and NIH3T3 cells infected with constructs containing this modified expression cassette revealed even more EGFP protein than in cells infected with the EGFP/Puro long construct. The “cleavage” of the T2A cassette appears to be very efficient, as almost no full-length EGFP-T2A-Puro fusion protein is detectable.

The functionality of the Puromycin resistance cassette in this modified construct was also evaluated in infected and Puromycin-selected 38B9 and NIH3T3 cells by flow cytometric analysis. Use of the modified selection cassette resulted in rapid and efficient accumulation of almost exclusively EGFP-positive cells, indicating sufficient amounts of translated PAC and functionality of the PAC expression cassette comparable to pBMN-I-EGFP/Puro. For a more accurate determination of PAC amounts, Western blot analysis should have been performed. Since the T2A constructs are not “true” fusion proteins, Western blot analysis with a GFP antibody could not be used in this case. Unfortunately, the only available anti-PAC antibody reported to work in Western blot analysis (clone 1H12L8, Thermo Fisher Scientific) did not show any signal even after several attempts. To obtain a rough estimate of the PAC amount, linker- and T2A-containing retroviral constructs with a C-terminal Myc tag were generated and PAC amounts analyzed in infected and selected cells by Western blot analysis. This experiment revealed that considerably more EGFP/Puro-Myc protein is present in the infected cells than T2A-generated Puro-Myc protein in both 38B9- and NIH3T3 cells. However, knowing the amount of PAC protein is of limited value because one is a fusion protein and the other is a T2A-processed protein with an additional N-terminal proline, which has been reported to sometimes destabilize T2A-processed proteins, resulting in increased protein degradation [22]. A direct comparison of the conferred PAC activity between the two cassettes could only be made by determining the enzymatic activity. However, since the selection efficiency of both constructs is very comparable, we conclude that even lower PAC amounts generated by EGFP-T2A-Puro constructs are sufficient for efficient inactivation of Puromycin.

Notably, Puromycin killing is most effective in rapidly dividing cells such as 38B9 because ongoing translation is a prerequisite for its mode of action. Adherent NIH3T3 cells and near-confluent cells therefore do not respond as quickly to Puromycin treatment as rapidly growing cells, but there is good selection and large enough fluorescence differences to separate infected from uninfected cells. If generally higher infection efficiencies are desired, robust cells can be infected on two consecutive days, resulting in more infected cells with slightly higher EGFP fluorescence intensities. Together with pBMN-I-TagBFP, pBMN-I-EGFP-T2A-Puro will be made available to the research community via Addgene.

## 5. Conclusions

In conclusion, the vector pBMN-I-EGFP-T2A-Puro is the construct of choice for successful stable retroviral infection, EGFP visualization, and Puromycin selection of mammalian suspension and adherent cells.

## Data Availability

The data presented in this study are available on request from the corresponding author.

## Supplementary Materials

The following supporting information can be downloaded at: https://www.mdpi.com/article/10.3390/biom14091131/s1, Figure S1: Vector map for pBMN-I-GFP; Figure S2: Vector map for pBMN-I-EGFP/Puro; Figure S3: Vector map for pBMN-I-TagBFP; Figure S4: Representative flow cytometric gating strategy for EGFP- and TagBFP fluorescence of 38B9 cells infected with different retroviral supernatants; Figure S5: Flow cytometric analyses for EGFP- and TagBFP fluorescence of NIH3T3 cells infected with different retroviral supernatants; Figure S6: Vector map for pBMN-I-EGFP/Puro long; Figure S7: Vector map for pBMN-I-EGFP/Puro Myc long; Figure S8: Quantification of reverse transcriptase activity in retroviral supernatants to determine relative retroviral titers; Figure S9: Cropped and uncropped images of Western blot analysis of EGFP proteins in wild-type- and infected 38B9 cells from Figure 2A; Figure S10: Cropped and uncropped images of Western blot analysis of EGFP proteins in wild-type- and infected NIH3T3 cells from Figure 2B; Figure S11: AlphaFold structure predictions of short and long EGFP/Puro fusion proteins and superposition of the two proteins; Figure S12: Flow cytometric analyses for EGFP fluorescence in 38B9 cells infected with pBMN-I-EGFP/Puro Myc long supernatant and subsequent Puromycin selection; Figure S13: Flow cytometric analyses for EGFP fluorescence in NIH3T3 cells infected with different retroviral supernatants and subsequent Puromycin selection; Figure S14: Vector map for pBMN-I-EGFP-T2A-Puro; Figure S15: Vector map for pBMN-I-EGFP-T2A-Puro Myc; Figure S16: Flow cytometric analyses for EGFP fluorescence of 38B9- and NIH3T3 cells infected with different retroviral supernatants; Figure S17: Flow cytometric analyses for EGFP fluorescence in NIH3T3 cells infected with different retroviral supernatants and subsequent Puromycin selection; Figure S18: Western blot analysis of Myc-tagged proteins in wild-type- and infected 38B9 cells; Figure S19: Western blot analysis of Myc-tagged proteins in wild-type- and infected NIH3T3 cells.

## Appendix A. Supplementary Methods

### Appendix A.1. Quantification of Reverse Transcriptase Activity by Real-Time PCR for Titration of Retroviral Vectors

Quantification of retroviruses in cell culture supernatants was done by measuring reverse transcriptase (RT) activity using the previously described SYBR Green I-based-PERT (SG-PERT) assay [20]. A total of 5 μL of viral supernatant or tenfold dilutions thereof up to 10^−7^ were incubated with 5 μL 2× concentrated lysis buffer (0.25% Triton X-100, 50 mM KCl, 100 mM Tris HCl pH7.4, 40% glycerol, 0.8 U/μL RiboLock RNase Inhibitor (Thermo Fisher Scientific)) for 10 min at room temperature. This mixture was then diluted with 90 μL of ultrapure water, and 9.6 μL was transferred to a 96-well PCR MicroAmp optical plate (Thermo Fisher Scientific) as input for the assay. Per well, 10.4 μL of the following reaction mix was used: 10 μL PowerTrack SYBR Green Mastermix (Thermo Fisher Scientific), 0.1 μL 10-fold diluted RNase Inhibitor, 0.1 μL of MS2 RNA (Roche, cat.# 10165948001), and 0.1 μL of each 100 μM primer MS2-F (TCCTGCTCAACTTCCTGTCGAG) and primer MS2-R (CACAGGTCAAACCTCCTAGGAATG). Each biological sample was analyzed in duplicates. Plates were read on an Applied Biosystems QuantStudio 1 Real-Time PCR System (Thermo Fisher Scientific), and melt curve analysis as well as cycle threshold levels were determined.

### Appendix A.2. Titration of Puromycin on 38B9- and NIH3T3 Cells

To establish the optimal Puromycin concentration for selection, various doses of Puromycin (Sigma-Aldrich, P4512, 10 mg/mL in H_2_O) were tested on 38B9- and NIH3T3 cells. A total of 2.5 × 10^4^ NIH3T3 cells were maintained in 1 mL DMEM complete medium in a 24-well plate and incubated overnight at 37 °C/7.5% CO_2_. Medium was replaced with fresh medium containing Puromycin at the final concentrations of 0, 1, 2, 3, 4, or 5 µg/mL. Viability was estimated the next day by visual inspection for dead cells under a bright field microscope. A total of 1.2 × 10^6^ 38B9 cells were maintained in 7 mL RPMI1640 complete medium in 25 cm^2^ flasks. Puromycin at the final concentrations of 0, 1, 2, 3, 4, or 5 µg/mL was added, and cell viability was estimated by measuring the forward and side scatter by flow cytometric analysis every day. After 3 days, cells received fresh Puromycin-containing medium. The optimal Puromycin concentration for both 38B9- and NIH3T3 cells was determined to be 5 µg/mL.
