# Supplementary material for: An Enhanced Retroviral Vector for Efficient Genetic Manipulation and Selection in Mammalian Cells
Source: Biomolecules. 2024 Sep 6;14(9):1131. doi: 10.3390/biom14091131 (PMC11430422; doi:10.3390/biom14091131)
Supplement: Supplementary file 1 [file biomolecules-14-01131-s001.zip › Supplemental_Figures_Legends_Triller_et_al.docx]

**Supplemental Figure S1| Vector map for pBMN-I-GFP.**

Vector map for plasmid pBMN-I-GFP (6353 bp, Addgene plasmid # 1736). Plasmid features: Moloney Murine Leukemia Virus 5‘ long terminal repeat (MoMuLV 5' LTR, bp 1 – 592), psi packaging element (psi, bp 593 – 1879), Encephalomyocarditis virus (EMCV) internal ribosome entry site (IRES, bp 1973 – 2472), Enhanced green fluorescent protein (EGFP, bp 2545 – 3264), Moloney Murine Leukemia Virus 3‘ long terminal repeat (MoMuLV 3' LTR, bp 3328 – 3919), pBR322 origin of replication (ori, bp 4205 – 4824), Ampicillin-resistance gene beta-lactamase (AmpR, bp 4932 – 5792). A selection of single-cutting restriction enzymes is shown. The plasmid map was created with Clone Manager 9 Professional Edition (Sci Ed Software, Westminster, CO, USA).

**Supplemental Figure S2| Vector map for pBMN-I-EGFP/Puro.**

Vector map for plasmid pBMN-I-EGFP/Puro (6965 bp, this work and [9]). Plasmid features: Moloney Murine Leukemia Virus 5‘ long terminal repeat (MoMuLV 5' LTR, bp 1 – 592), psi packaging element (psi, bp 593 – 1879), Encephalomyocarditis virus (EMCV) internal ribosome entry site (IRES, bp 1973 – 2472), Enhanced green fluorescent protein (EGFP, bp 2545 – 3261), linker peptide (linker, bp 3262 – 3291), Puromycin-N-acetyltransferase (PuroR, bp 3292 – 3891), Moloney Murine Leukemia Virus 3‘ long terminal repeat (MoMuLV 3' LTR, bp 3940 – 4531), pBR322 origin of replication (ori, bp 4817 – 5436), Ampicillin-resistance gene beta-lactamase (AmpR, bp 5544 – 6404). A selection of single-cutting restriction enzymes is shown. The plasmid map was created with Clone Manager 9 Professional Edition (Sci Ed Software, Westminster, CO, USA).

**Supplemental Figure S3| Vector map for pBMN-I-TagBFP.**

Vector map for plasmid pBMN-I-TagBFP (6338 bp, this work). Plasmid features: Moloney Murine Leukemia Virus 5‘ long terminal repeat (MoMuLV 5' LTR, bp 1 – 592), psi packaging element (psi, bp 593 – 1879), Encephalomyocarditis virus (EMCV) internal ribosome entry site (IRES, bp 1973 – 2472), Tag blue fluorescent protein (TagBFP, bp 2545 – 3249), Moloney Murine Leukemia Virus 3‘ long terminal repeat (MoMuLV 3' LTR, bp 3313 – 3904), pBR322 origin of replication (ori, bp 4190 – 4809), Ampicillin-resistance gene beta-lactamase (AmpR, bp 4917 – 5777). A selection of single-cutting restriction enzymes is shown. The plasmid map was created with Clone Manager 9 Professional Edition (Sci Ed Software, Westminster, CO, USA).

**Supplemental Figure S4| Representative flow cytometric gating strategy for EGFP- and TagBFP fluorescence of 38B9 cells infected with different retroviral supernatants.**

Exemplary raw data for some analyses shown in Figure 1. 38B9 cells were **A)** mock infected or infected with retroviral supernatants derived from **B)** pBMN-I-GFP or **C)** pBMN-I-TagBFP, and incubated for one day before flow cytometric analysis to determine infection efficiencies. For the hierarchical sequences of analysis (i.e. gating strategy), the scattered light intensity was measured by the forward scatter- (FS INT) and sideward scatter (SS INT) detectors, and viable cells are displayed in gate “A” (1^st^ column). A plot of the FS peak signal (FS PEAK) against the area based FS intensity (FS INT) allowed to exclude multimeric cell agglomerates, and single cells are displayed in gate “B” (2^nd^ column). Cells present in gates “A” and “B” (COUNT) are then displayed in histogram plots for EGFP- [FI (EGFP), gate “I”, 3^rd^ column] or BFP- [FI (BFP), gate “J”, 4^th^ column] fluorescence intensities. Percentages of gated cells are indicated in the graphs. Data were collected with a Gallios flow cytometer and analyzed with Kaluza software. Results are representative of three independent experiments.

**Supplemental Figure S5| Flow cytometric analyses for EGFP- and TagBFP fluorescence of NIH3T3 cells infected with different retroviral supernatants.**

NIH3T3 cells were **A)** mock infected or infected with different retroviral supernatants derived from **B)** pBMN-I-GFP, **C)** pBMN-I-TagBFP, **D)** pBMN-I-EGFP/Puro, and **E)** pBMN-I-EGFP/Puro long, and incubated for one day before flow cytometric analysis to determine infection efficiencies. The flow cytometric gating strategy is identical to that shown in Supplemental Figure 4. Cells (COUNT) are shown in histogram plots for EGFP- [FI (EGFP), gate “I”, upper row] or BFP- [FI (BFP), gate “H”, bottom row] fluorescence intensities. Percentages of gated cells are shown in the graphs. Data were collected using a Gallios flow cytometer and analyzed using Kaluza software. The results are representative of three independent experiments.

**Supplemental Figure S6| Vector map for pBMN-I-EGFP/Puro long.**

Vector map for plasmid pBMN-I-EGFP/Puro long (6981 bp, this work). Plasmid features: Moloney Murine Leukemia Virus 5‘ long terminal repeat (MoMuLV 5' LTR, bp 1 – 592), psi packaging element (psi, bp 593 – 1879), Encephalomyocarditis virus (EMCV) internal ribosome entry site (IRES, bp 1972 – 2471), Enhanced green fluorescent protein (EGFP, bp 2544 – 3260), linker peptide (linker, bp 3261 – 3290), Puromycin-N-acetyltransferase (PuroR long, bp 3291 – 3908), Moloney Murine Leukemia Virus 3‘ long terminal repeat (MoMuLV 3' LTR, bp 3956 – 4547), pBR322 origin of replication (ori, bp 4833 – 5452), Ampicillin-resistance gene beta-lactamase (AmpR, bp 5560 – 6420). A selection of single-cutting restriction enzymes is shown. The plasmid map was created with Clone Manager 9 Professional Edition (Sci Ed Software, Westminster, CO, USA).

**Supplemental Figure S7| Vector map for pBMN-I-EGFP/Puro Myc long.**

Vector map for plasmid pBMN-I-EGFP/Puro Myc long (7011 bp, this work). Plasmid features: Moloney Murine Leukemia Virus 5‘ long terminal repeat (MoMuLV 5' LTR, bp 1 – 592), psi packaging element (psi, bp 593 – 1879), Encephalomyocarditis virus (EMCV) internal ribosome entry site (IRES, bp 1972 – 2471), Enhanced green fluorescent protein (EGFP, bp 2544 – 3260), linker peptide (linker, bp 3261 – 3290), Puromycin-N-acetyltransferase (PuroR long, bp 3291 – 3905), Myc tag (Myc, bp 3906 – 3935), Moloney Murine Leukemia Virus 3‘ long terminal repeat (MoMuLV 3' LTR, bp 3986 – 4577), pBR322 origin of replication (ori, bp 4863 – 5482), Ampicillin-resistance gene beta-lactamase (AmpR, bp 5590 – 6450). A selection of single-cutting restriction enzymes is shown. The plasmid map was created with Clone Manager 9 Professional Edition (Sci Ed Software, Westminster, CO, USA).

**Supplemental Figure S8| Quantification of reverse transcriptase activity in retroviral supernatants to determine relative retroviral titers.**To determine relative retroviral titers in the cell culture supernatants used for infection, reverse transcriptase (RT) activity was measured using the SG-PERT assay. Viral supernatants of wild-type Platinum-E cells, mock transfected Platinum-E cells, cells transfected with retroviral constructs pBMN-I-GFP, pBMN-I-TagBFP, pBMN-I-EGFP/Puro, pBMN-I-EGFP/Puro long, pBMN-I-EGFP/Puro Myc long, pBMN-I-EGFP-T2A-Puro, or pBMN-I-EGFP-T2A-Puro Myc and seven tenfold dilutions thereof (log concentration 10^0^ – 10^-7^) were added to 2x concentrated lysis buffer and incubated at room temperature. After dilution with ultrapure water, parts of the lysates were transferred to a 96-well PCR MicroAmp optical plate and 2x PowerTrack SYBR Green Mastermix as well as RNase inhibitor, Phage MS2 RNA and MS2-specific forward and reverse primers were added. A quantitative RT real time PCR analysis on an Applied Biosystems QuantStudio 1 Real-Time PCR System was performed, followed by melting curve analysis and determination of the cycle threshold (Ct) values. **A)** Each biological sample was analyzed in duplicates and mean Ct values are depicted. PCR products were analyzed on an 1.5% agarose gel stained with Ethidium bromide (data not shown). Observed Ct values for wild-type and mock supernatants were from nonspecific amplification and did not yield the expected PCR product of 112 bp. Results are representative of three independent experiments. **B)** Results of A) depicted as correlation between the input level of retroviral supernatants and Ct values.

**Supplemental Figure S9| Cropped and uncropped images of Western blot analysis of EGFP proteins in wild-type- and infected 38B9 cells from Figure 2A.**

Shown are **A)** the cropped and **B)** uncropped images of the Western blot analysis depicted in Figure 2A. Please refer to the legend of Figure 2 for further details.

**Supplemental Figure S10| Cropped and uncropped images of Western blot analysis of EGFP proteins in wild-type- and infected NIH3T3 cells from Figure 2B.**

Shown are **A)** the cropped and **B)** uncropped images of the Western blot analysis depicted in Figure 2B. Please refer to the legend of Figure 2 for further details.

**Supplemental Figure S11| Structural alignment of AlphaFold predictions of short and long EGFP/Puro fusion proteins.**

An AlphaFold Colab notebook was used to predict the protein structures for **A)**, **B)** EGFP/Puro (orange) and **D)**, **E)** EGFP/Puro long (blue). The resulting PDB files were used for the overlay of both proteins [**C)**, **F)**] using the ”Pairwise Structure Alignment” tool of the RCSB Protein Data Bank. Displayed are the structures of the single proteins and the overlay of both both proteins from two different angels (A-C and D-F) The coordinates in the lower left corner of each structure indicate the orientation.

**Supplemental Figure S12| Flow cytometric analyses for EGFP fluorescence in 38B9 cells infected with pBMN-I-EGFP/Puro Myc long supernatant and subsequent Puromycin selection.**

38B9 cells were infected with retroviral supernatants derived from **A)** mock transfected- or **B)** pBMN-I-EGFP/Puro Myc long transfected Platinum-E cells. Puromycin was added at 5 µg/mL 24 hours after infection. EGFP fluorescence intensities to determine infection efficiency were measured by flow cytometry on the day of infection, one day after infection, and every other day for 5 days. Data acquisition and gating strategy were identical to those described in Supplemental figure 4. Data of mock infection are identical to those in Figure 3. EGFP fluorescence intensities of live and single cells are presented as overlay histograms, and relative cell numbers of each measurement are normalized and presented as %Max. The results are representative of three independent experiments.

**Supplemental Figure S13| Flow cytometric analyses for EGFP fluorescence in NIH3T3 cells infected with different retroviral supernatants and subsequent Puromycin selection.**

NIH3T3 cells were infected with retroviral supernatants derived from **A)** mock transfected Platinum-E cells, **B)** pBMN-I-EGFP/Puro or **C)** pBMN-I-EGFP/Puro long. Puromycin was added at 5 µg/mL 24 hours after infection. EGFP fluorescence intensities to determine infection efficiency were measured by flow cytometry on the day of infection, one day after infection, and every other day for 5 days. Data acquisition and gating strategy were identical to those described in Supplemental figure 4. EGFP fluorescence intensities of live and single cells are presented as overlay histograms, and relative cell numbers of each measurement are normalized and presented as %Max. The results are representative of two independent experiments.

**Supplemental Figure S14| Vector map for pBMN-I-EGFP-T2A-Puro.**

Vector map for plasmid pBMN-I-EGFP-T2A-Puro (6987 bp, this work). Plasmid features: Moloney Murine Leukemia Virus 5‘ long terminal repeat (MoMuLV 5' LTR, bp 1 – 592), psi packaging element (psi, bp 593 – 1879), Encephalomyocarditis virus (EMCV) internal ribosome entry site (IRES, bp 1971 – 2470), Enhanced green fluorescent protein (EGFP, bp 2543 – 3259), T2A self-cleaving peptide (T2A, bp 3260 – 3313), Puromycin-N-acetyltransferase (PuroR, bp 3314 – 3913), Moloney Murine Leukemia Virus 3‘ long terminal repeat (MoMuLV 3' LTR, bp 3962 – 4553), pBR322 origin of replication (ori, bp 4839 – 5458), Ampicillin-resistance gene beta-lactamase (AmpR, bp 5566 – 6426). A selection of single-cutting restriction enzymes is shown. The plasmid map was created with Clone Manager 9 Professional Edition (Sci Ed Software, Westminster, CO, USA).

**Supplemental Figure S15| Vector map for pBMN-I-EGFP-T2A-Puro Myc.**

Vector map for plasmid pBMN-I-EGFP-T2A-Puro Myc (7017 bp, this work). Plasmid features: Moloney Murine Leukemia Virus 5‘ long terminal repeat (MoMuLV 5' LTR, bp 1 – 592), psi packaging element (psi, bp 593 – 1879), Encephalomyocarditis virus (EMCV) internal ribosome entry site (IRES, bp 1971 – 2470), Enhanced green fluorescent protein (EGFP, bp 2543 – 3259), T2A self-cleaving peptide (T2A, bp 3260 – 3313), Puromycin-N-acetyltransferase (PuroR, bp 3314 – 3910), Myc tag (Myc, bp 3911 – 3943), Moloney Murine Leukemia Virus 3‘ long terminal repeat (MoMuLV 3' LTR, bp 3992 – 4583), pBR322 origin of replication (ori, bp 4869 – 5488), Ampicillin-resistance gene beta-lactamase (AmpR, bp 5596 – 6456). A selection of single-cutting restriction enzymes is shown. The plasmid map was created with Clone Manager 9 Professional Edition (Sci Ed Software, Westminster, CO, USA).

**Supplemental Figure S16| Flow cytometric analyses for EGFP fluorescence of 38B9- and NIH3T3 cells infected with different retroviral supernatants.**

38B9- (upper panel) or NIH3T3 cells (bottom panel) were **A)** mock infected or infected with retroviral supernatants derived from **B)** pBMN-I-EGFP-T2A-Puro, or **C)** pBMN-I-EGFP-T2A-Puro Myc, and incubated for one day before flow cytometric analysis to determine infection efficiencies. A detailed gating and analysis strategy is shown in Supplemental figure 4. Cells (COUNT) are shown in histogram plots for EGFP- [FI (EGFP), gate “I”, upper row] fluorescence intensities. Percentages of gated cells are shown in the graphs. Data were collected using a Gallios flow cytometer and analyzed using Kaluza software. The results are representative of three independent experiments.

**Supplemental Figure S17| Flow cytometric analyses for EGFP fluorescence in NIH3T3 cells infected with different retroviral supernatants and subsequent Puromycin selection.**

NIH3T3 cells were infected with retroviral supernatants derived from **A)** mock transfected Platinum-E cells, **B)** pBMN-I-EGFP-T2A-Puro or **C)** pBMN-I-EGFP-T2A-Puro Myc. Puromycin was added at 5 µg/mL 24 hours after infection,. EGFP fluorescence intensities to determine infection efficiency were measured by flow cytometry on the day of infection, one day after infection, and every other day for 5 days. Data acquisition and gating strategy were identical to those described in Figure 1. EGFP fluorescence intensities of live and single cells are presented as overlay histograms, and relative cell numbers of each measurement are normalized and presented as %Max. The results are representative of three independent experiments.

**Supplemental Figure S18| Western blot analysis of Myc-tagged proteins in wild-type- and infected 38B9 cells.**

**A)** Cell lysates from 1x10^6^ uninfected and unselected 38B9 cells (“WT”) or from 1x10^6^ 38B9 cells infected with retroviral supernatant from the respective vectors pBMN-I-EGFP-T2A-Puro (“EGFP-T2A-Puro”), pBMN-I-EGFP/Puro (“EGFP/Puro”), pBMN-I-EGFP/Puro long (“EGFP/Puro long”), pBMN-I-EGFP/Puro Myc long (“EGFP/Puro Myc long”) or pBMN-I-EGFP-T2A-Puro Myc (“EGFP-T2A-Puro Myc”) and Puromycin-selected for 5 days were reduced, separated by 13.5% SDS-PAGE, and transferred to a nitrocellulose membrane. The membrane was blocked, stained with mouse anti-Myc hybridoma supernatant and developed with an appropriate HRP-conjugated secondary antibody using the ECL method. The loading of the same cell equivalents was assessed with polyclonal rabbit antibodies against beta-actin (bottom panel). Dashed arrows indicate unspecific binding of the anti-Myc hybridoma supernatant (upper panel) or signals from previous probing with the Myc antibody (bottom panel). Results represent one of two independent experiments. **B)** Uncropped images of the Western blot analysis depicted in Supplemental Figure S18A.

**Supplemental Figure S19| Western blot analysis of Myc-tagged proteins in wild-type- and infected NIH3T3 cells.**

**A)** Cell lysates from 1x10^6^ uninfected and unselected NIH3T3 cells (“WT”) or from 1x10^6^ NIH3T3 cells infected with retroviral supernatant from the respective vectors pBMN-I-EGFP-T2A-Puro (“EGFP-T2A-Puro”), pBMN-I-EGFP/Puro (“EGFP/Puro”), pBMN-I-EGFP/Puro long (“EGFP/Puro long”), pBMN-I-EGFP/Puro Myc long (“EGFP/Puro Myc long”) or pBMN-I-EGFP-T2A-Puro Myc (“EGFP-T2A-Puro Myc”) and Puromycin-selected for 5 days were reduced, separated by 13.5% SDS-PAGE, and transferred to a nitrocellulose membrane. The membrane was blocked, stained with mouse anti-Myc hybridoma supernatant and developed with an appropriate HRP-conjugated secondary antibody using the ECL method. The loading of the same cell equivalents was assessed with polyclonal rabbit antibodies against beta-actin (bottom panel). Dashed arrows indicate unspecific binding of the anti-Myc hybridoma supernatant (upper panel) or signals from previous probing with the Myc antibody (bottom panel). Results represent one of two independent experiments. **B)** Uncropped images of the Western blot analysis depicted in Supplemental Figure S19A.
