# Supplementary material for: An Enhanced Retroviral Vector for Efficient Genetic Manipulation and Selection in Mammalian Cells
Source: Biomolecules. 2024 Sep 6;14(9):1131. doi: 10.3390/biom14091131 (PMC11430422; doi:10.3390/biom14091131)

## Slide 1
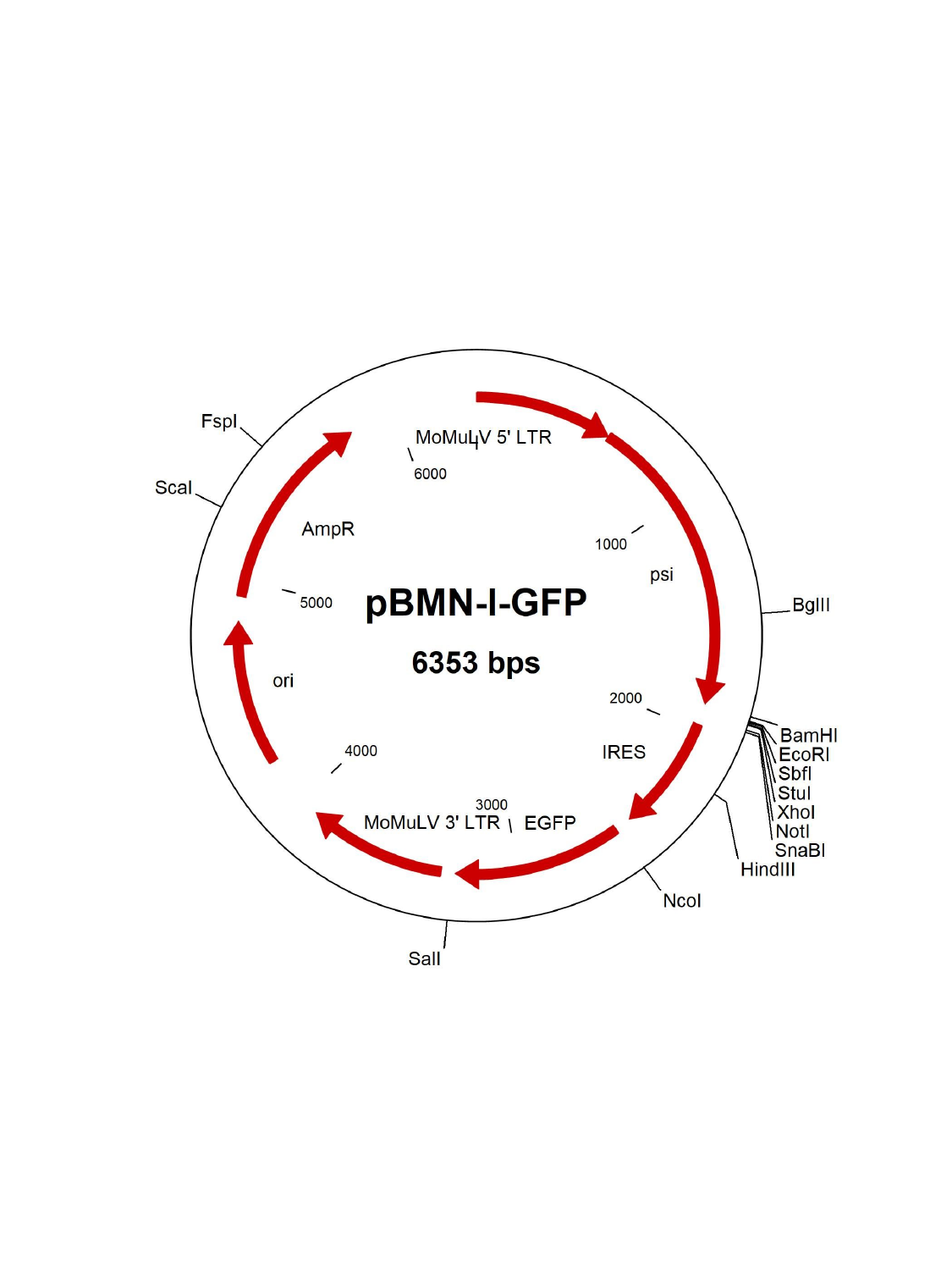

## Slide 2
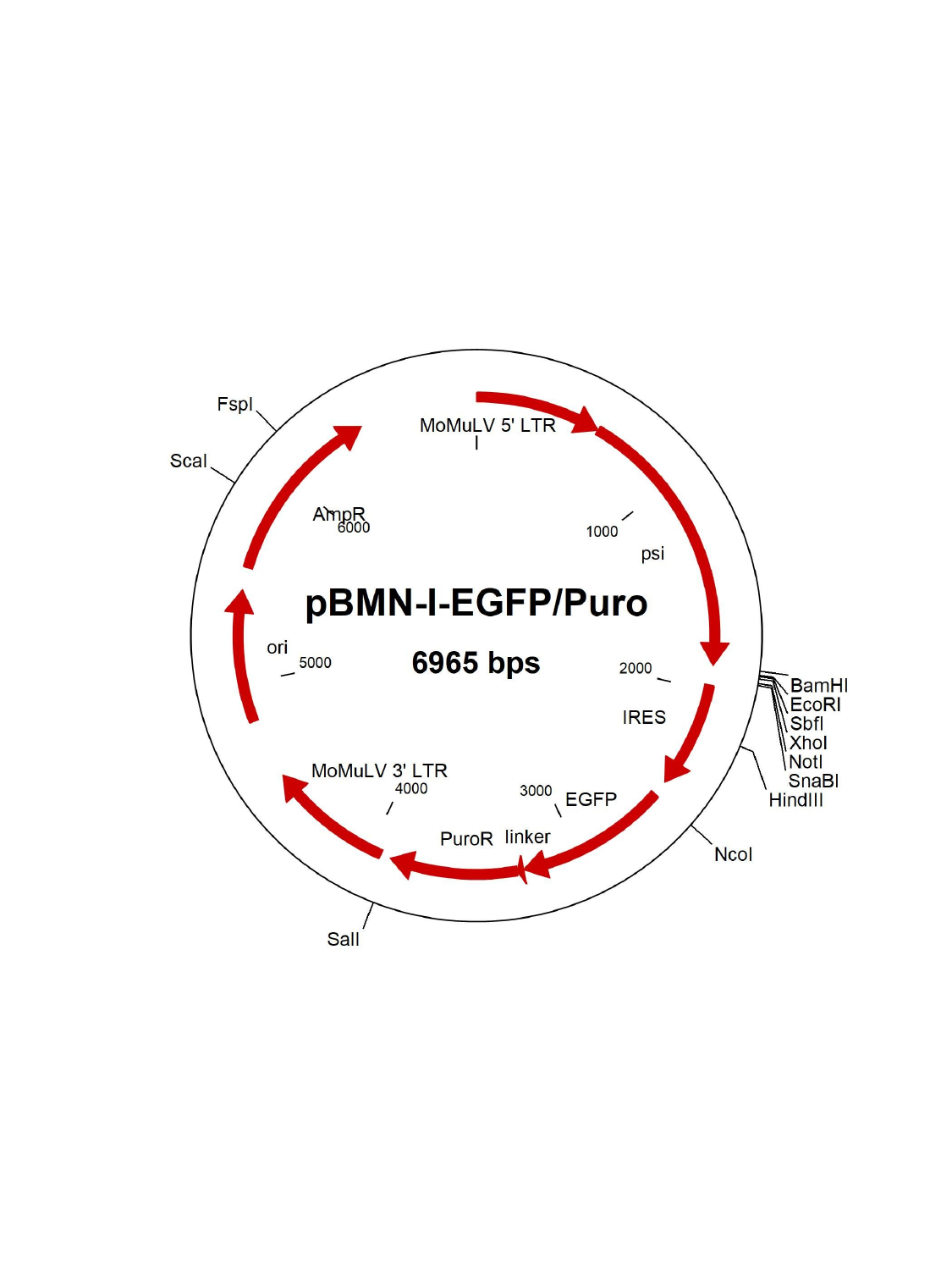

## Slide 3
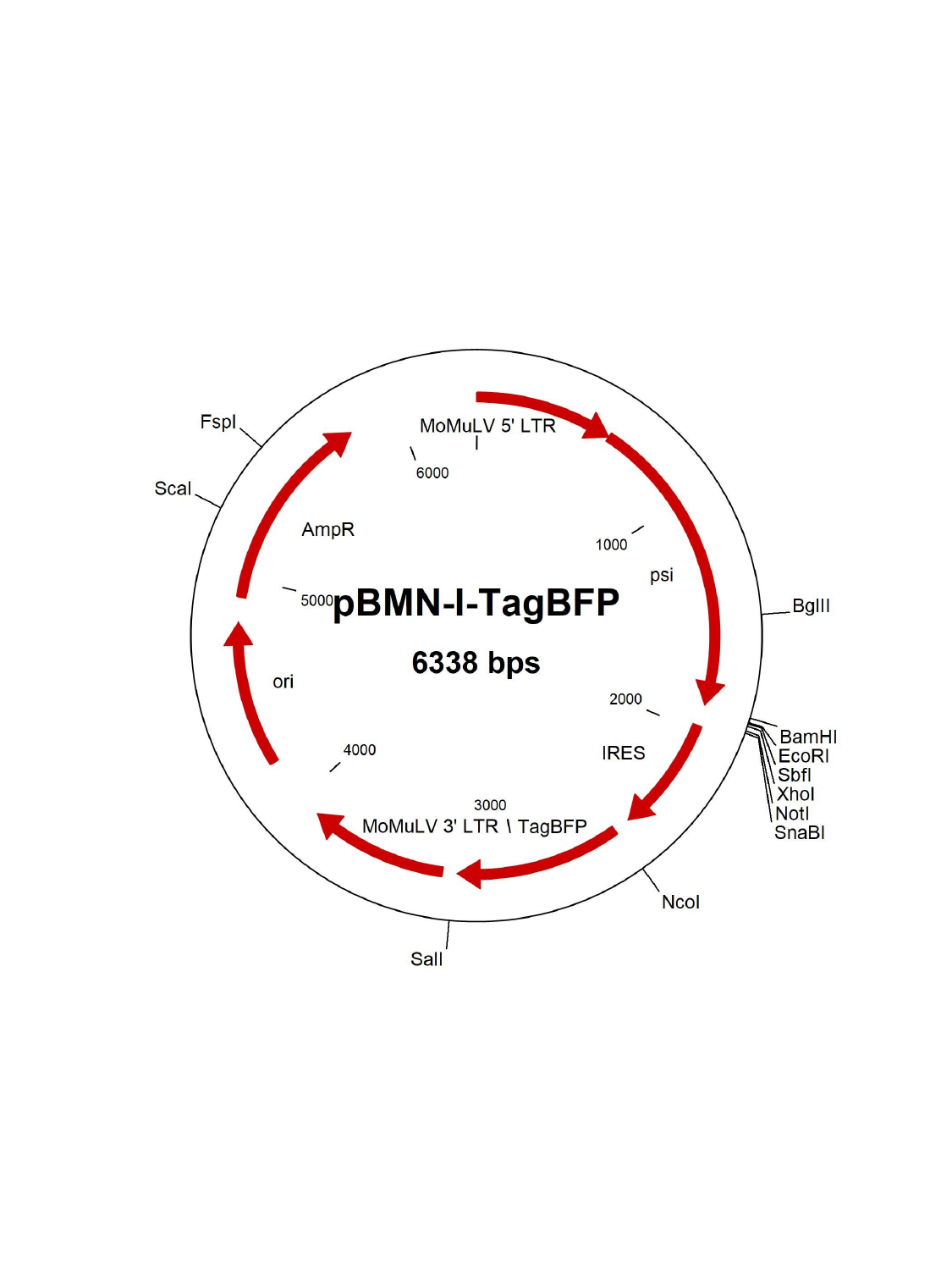

## Slide 4
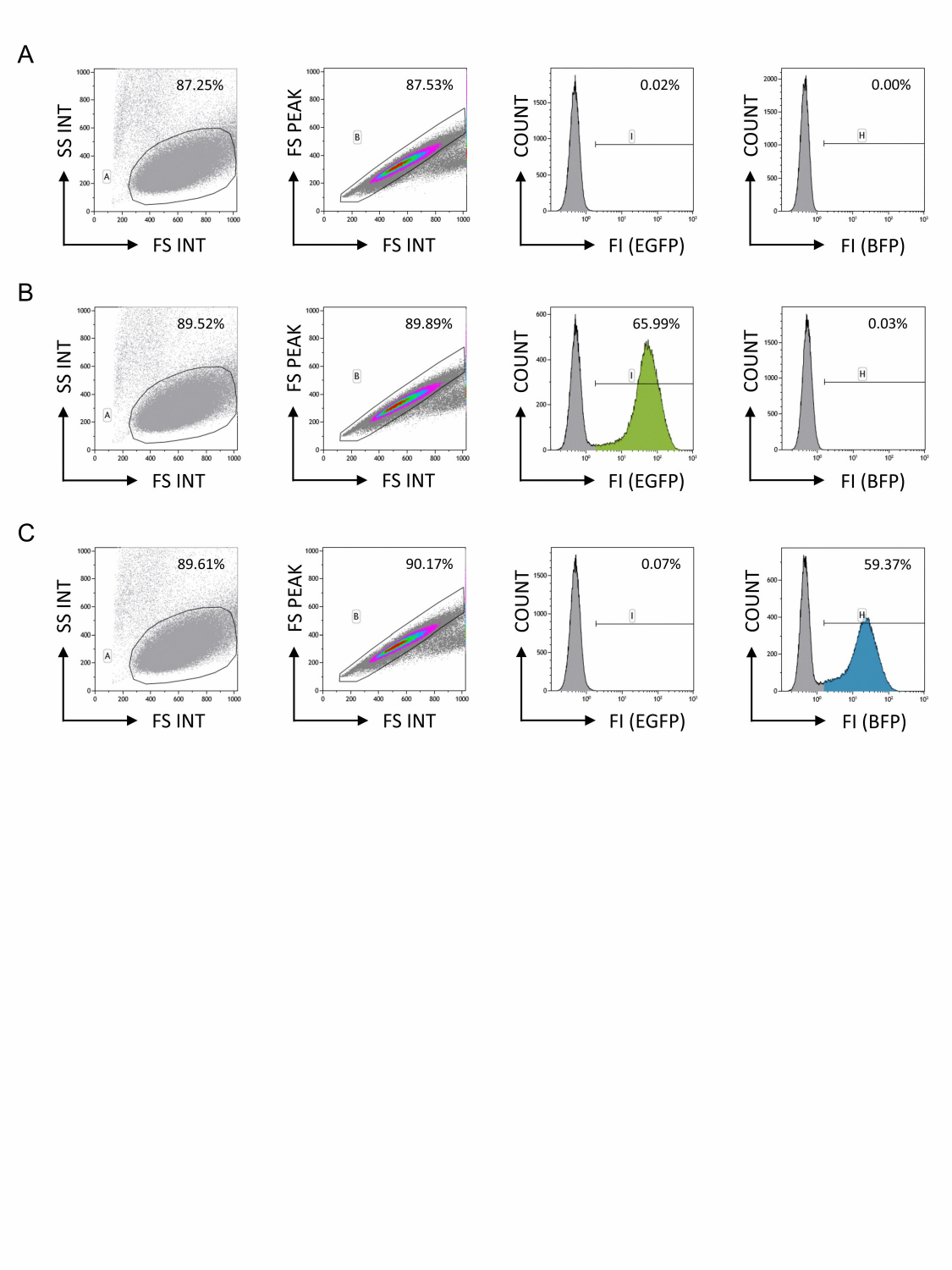

## Slide 5
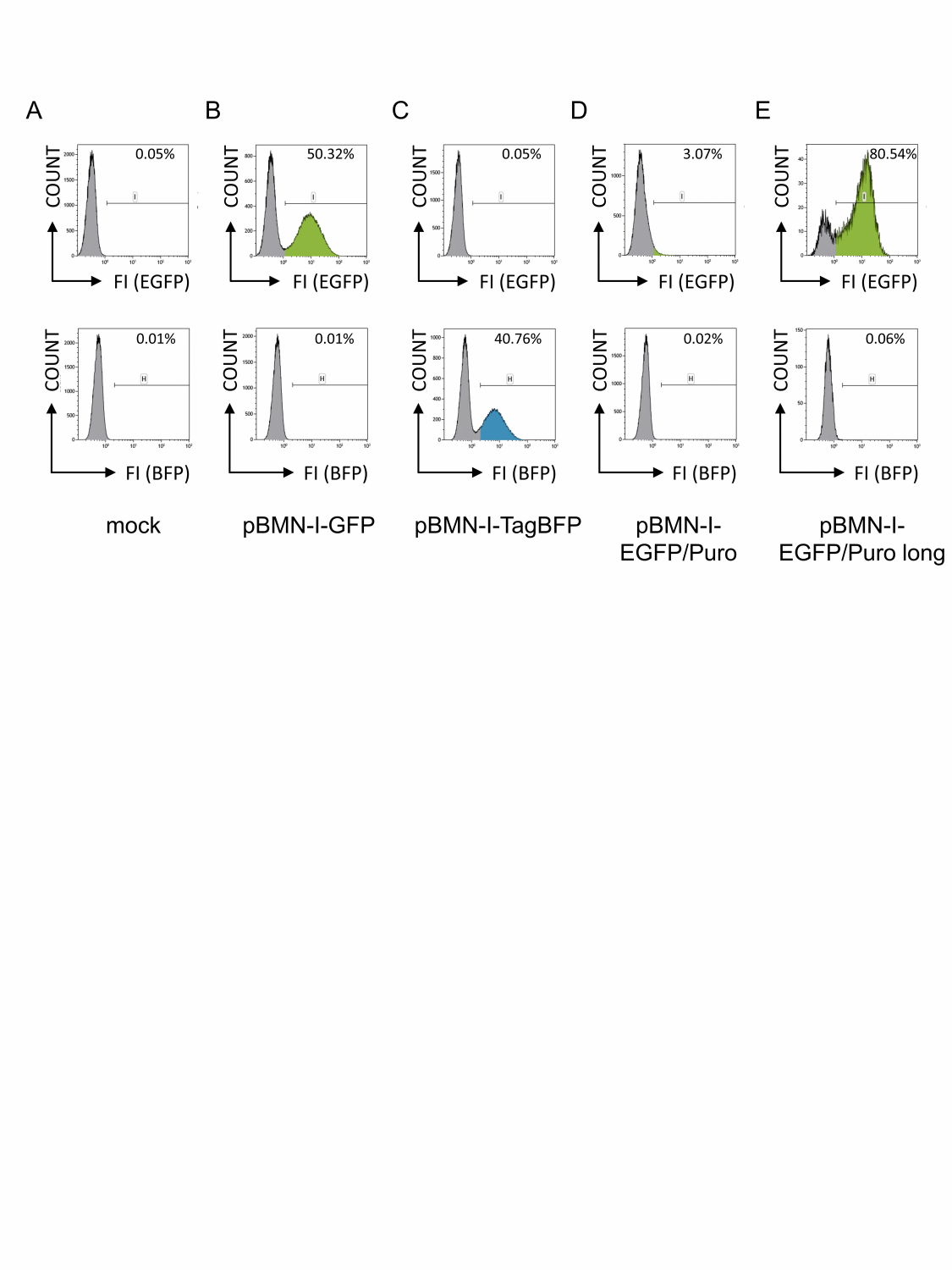

## Slide 6
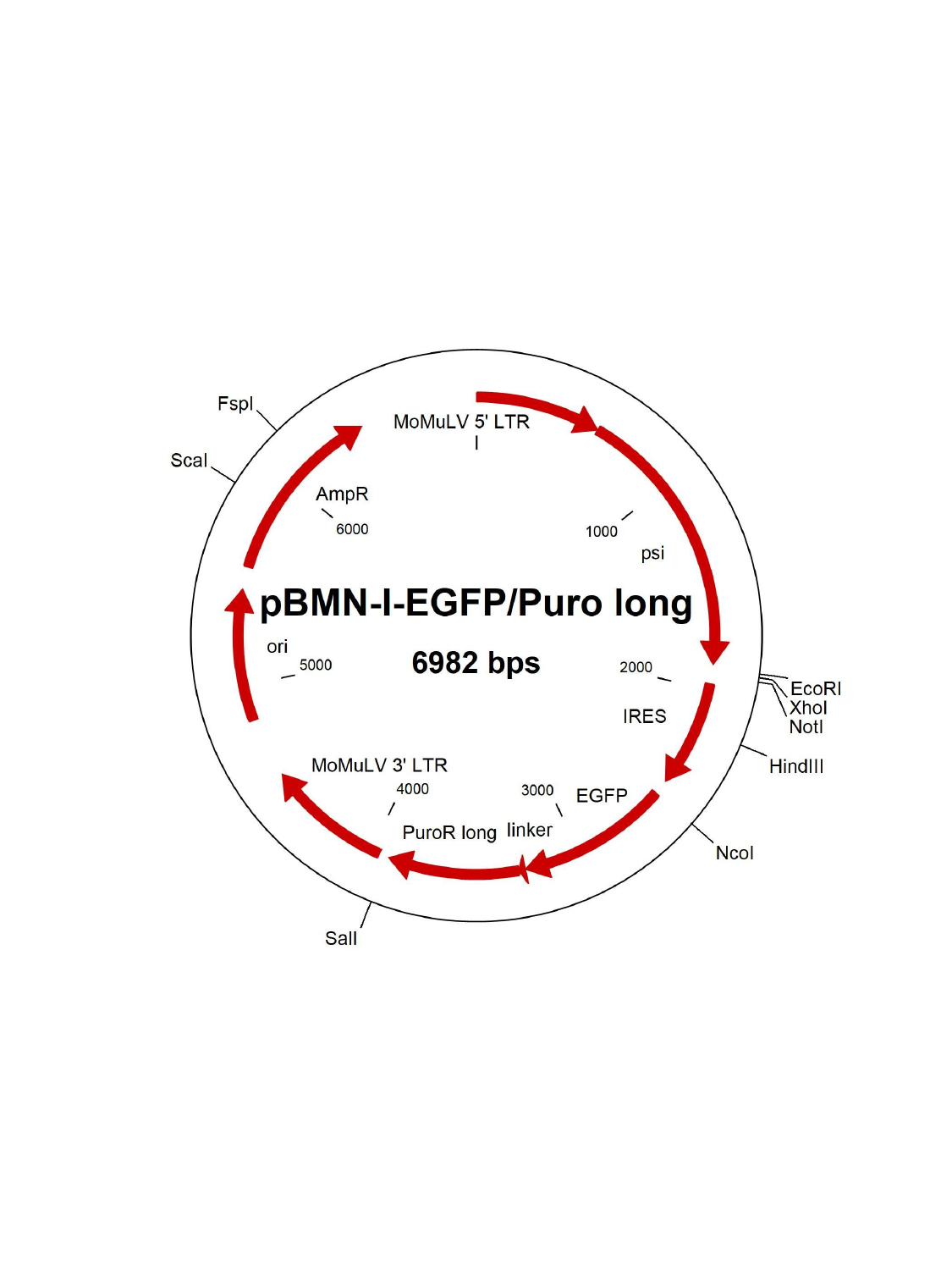

## Slide 7
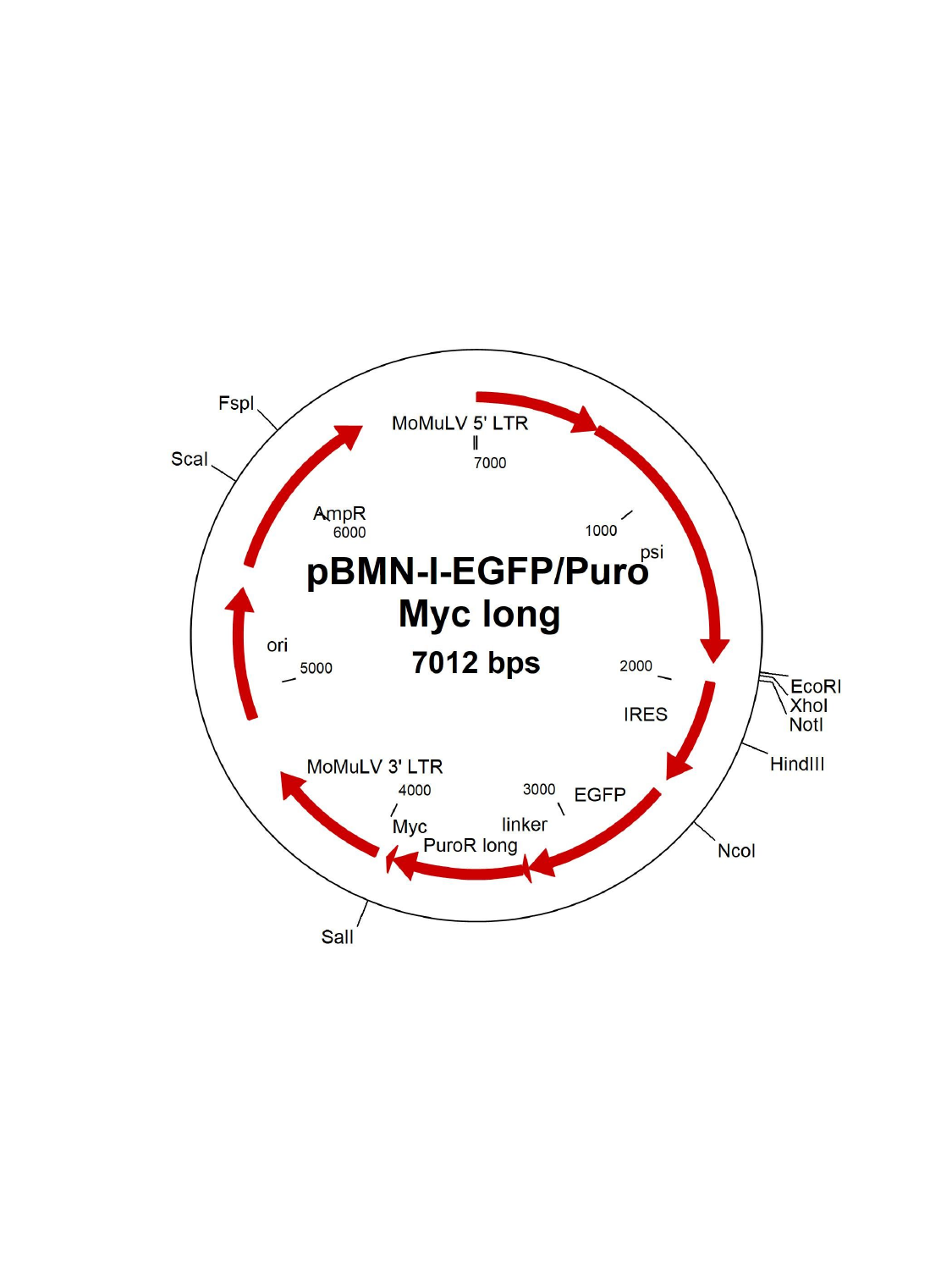

## Slide 8
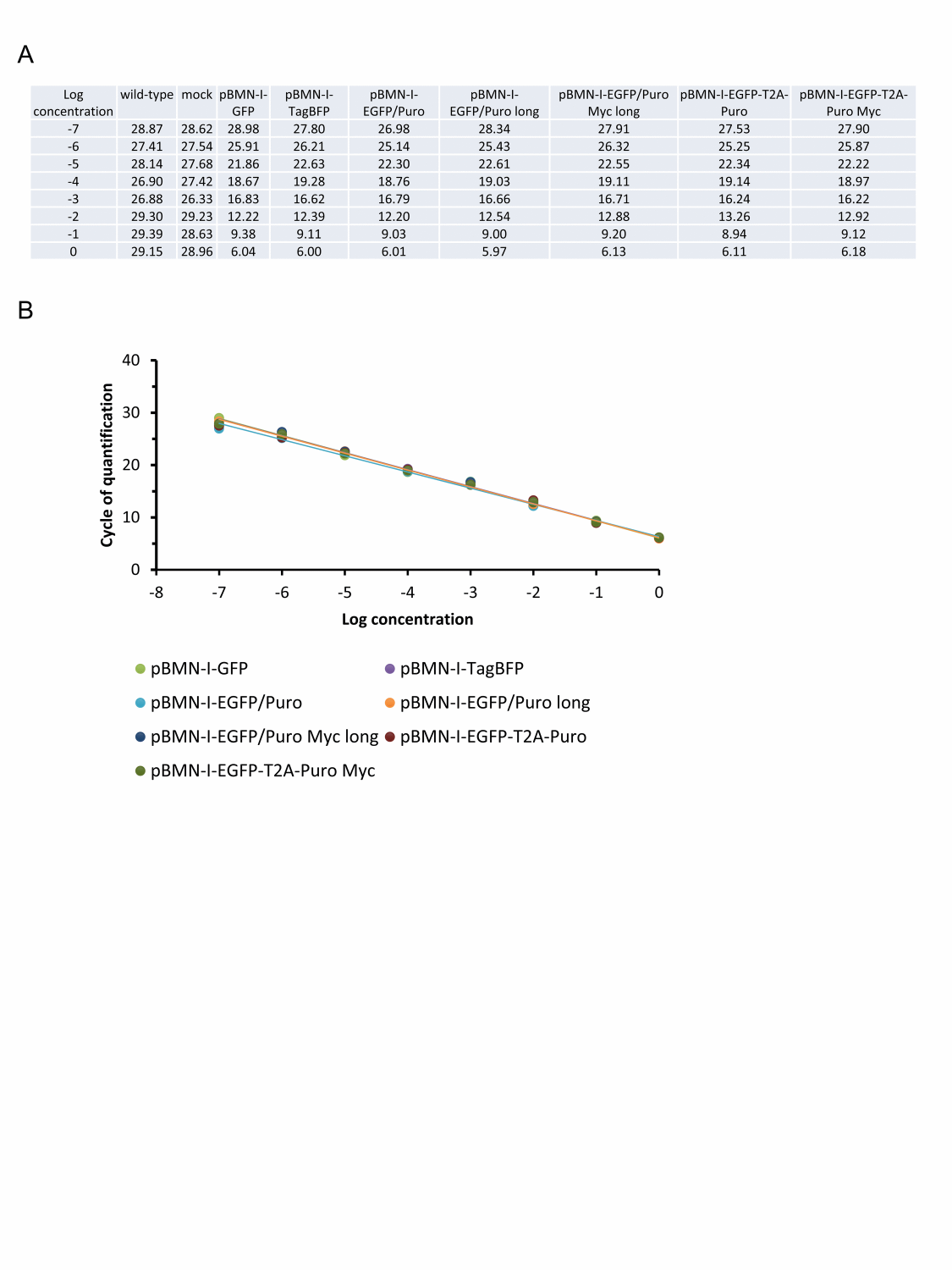

## Slide 9
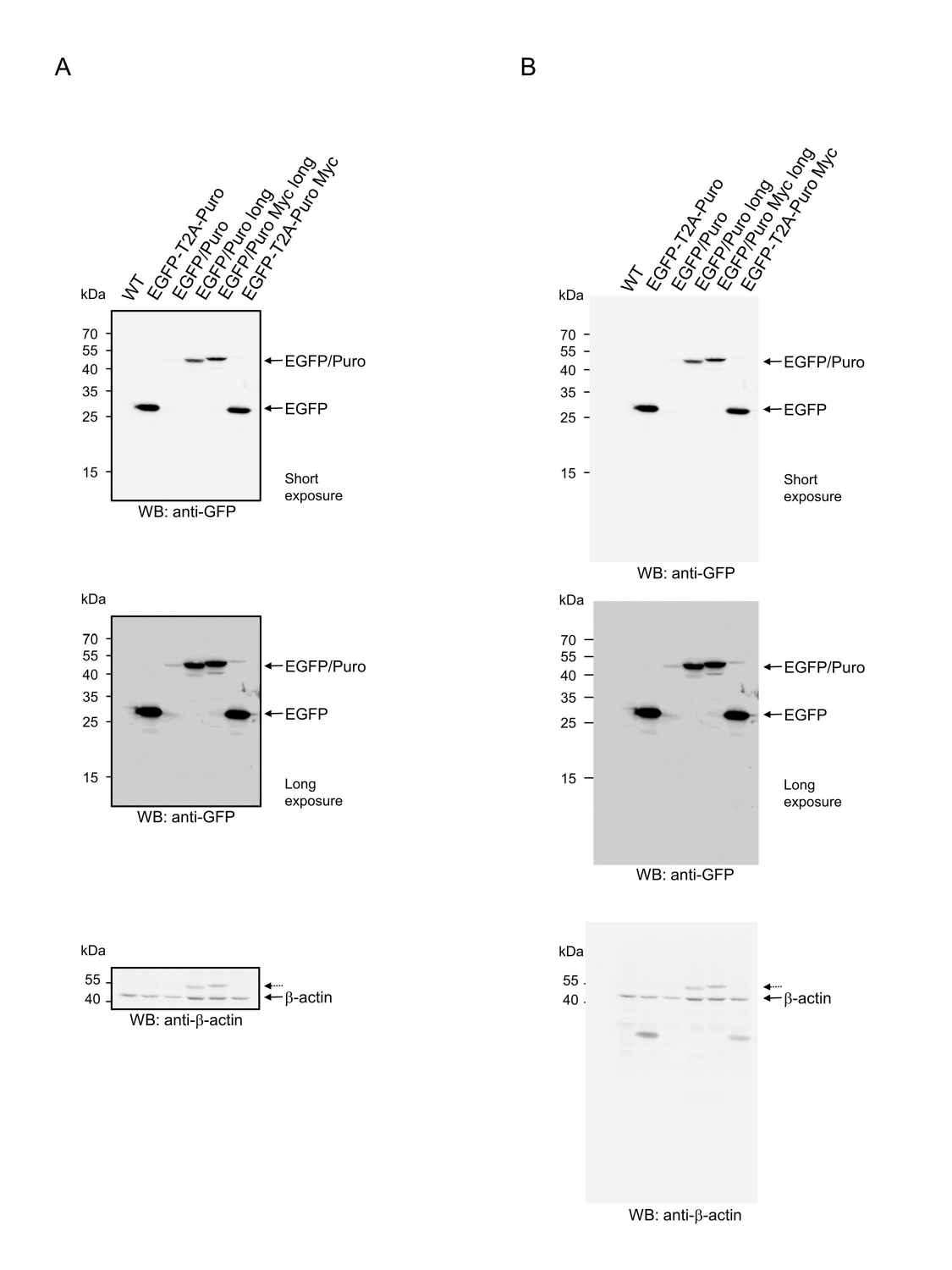

## Slide 10
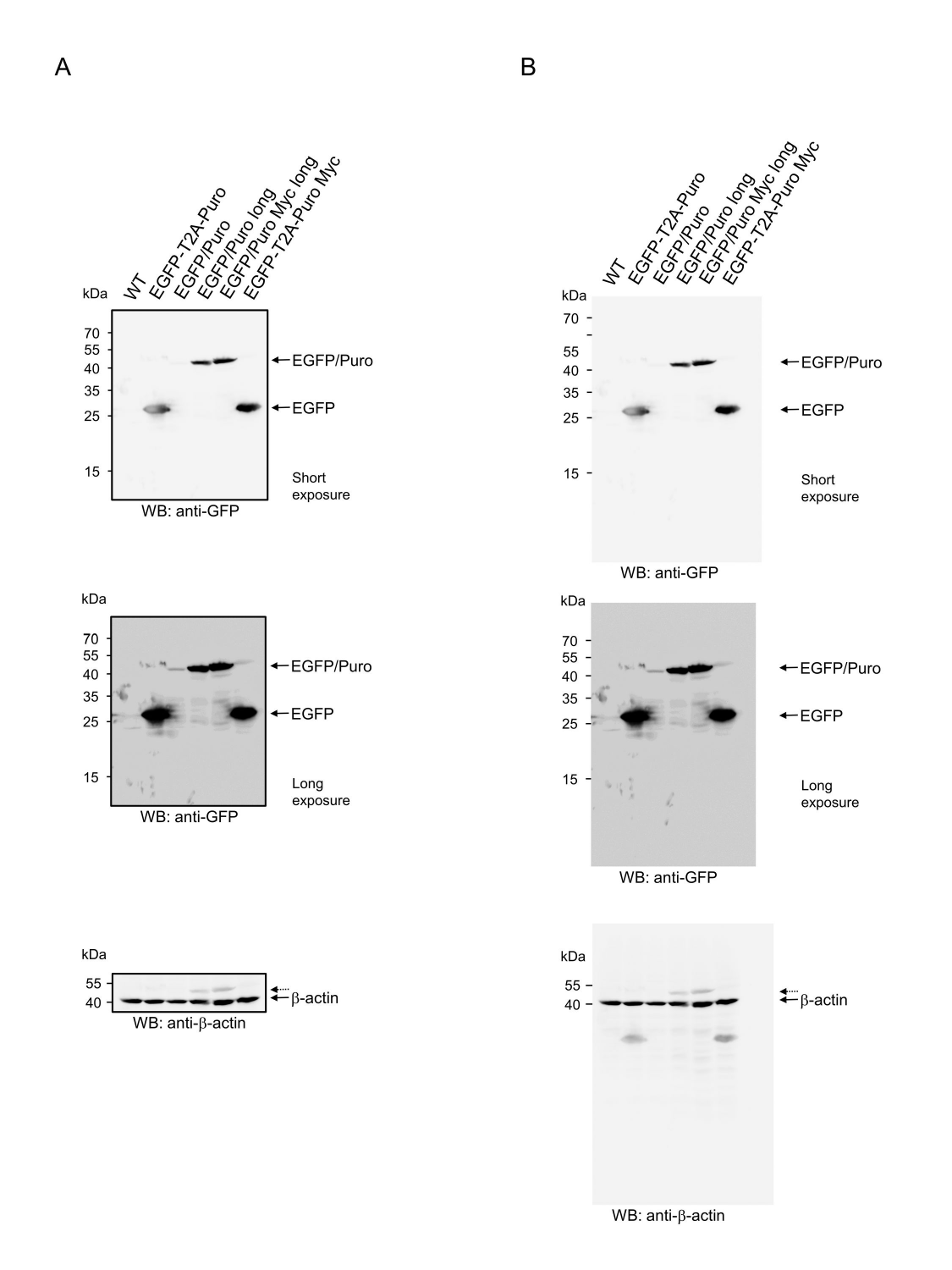

## Slide 11
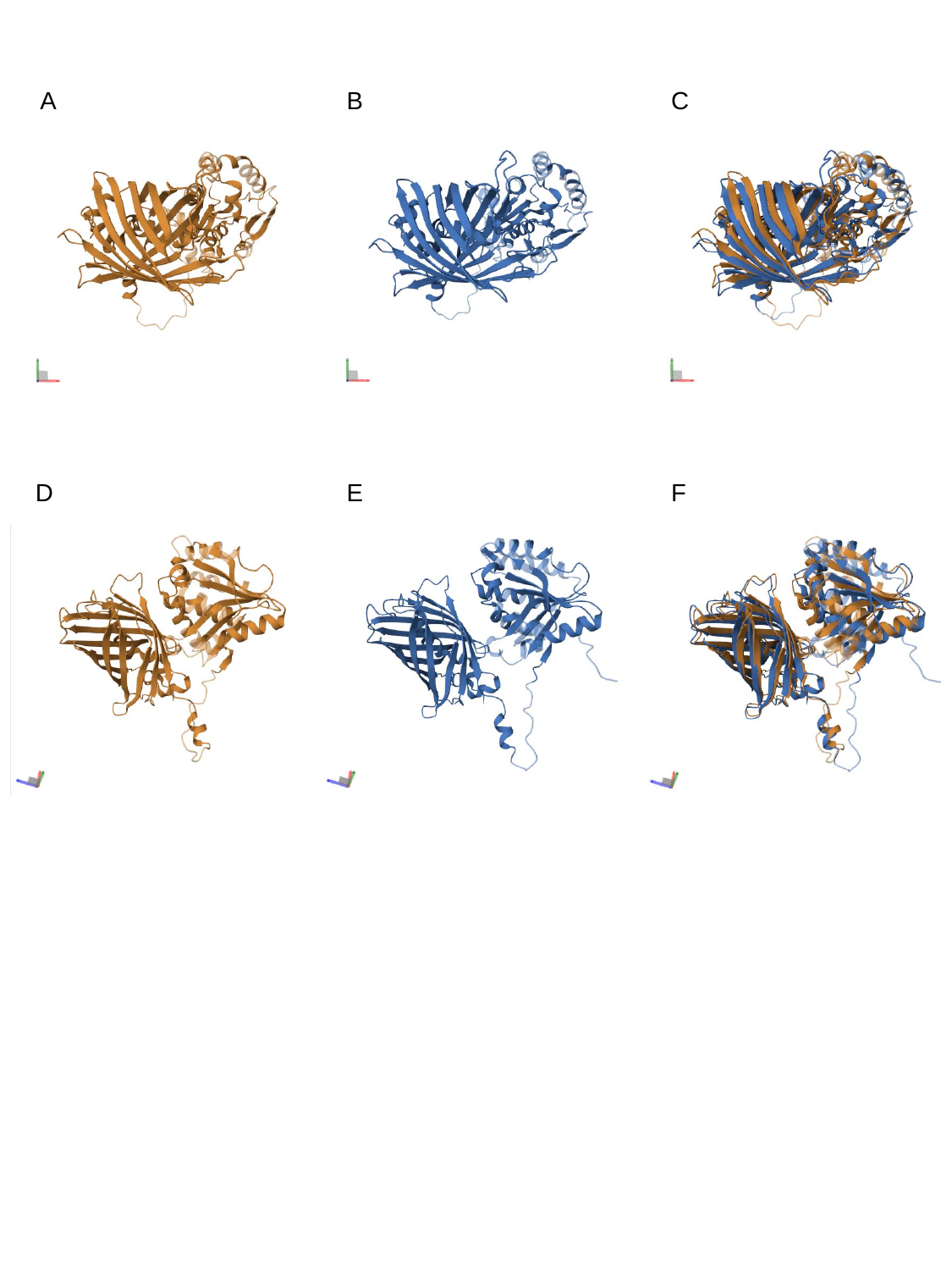

A
B
C
D
E
F

## Slide 12
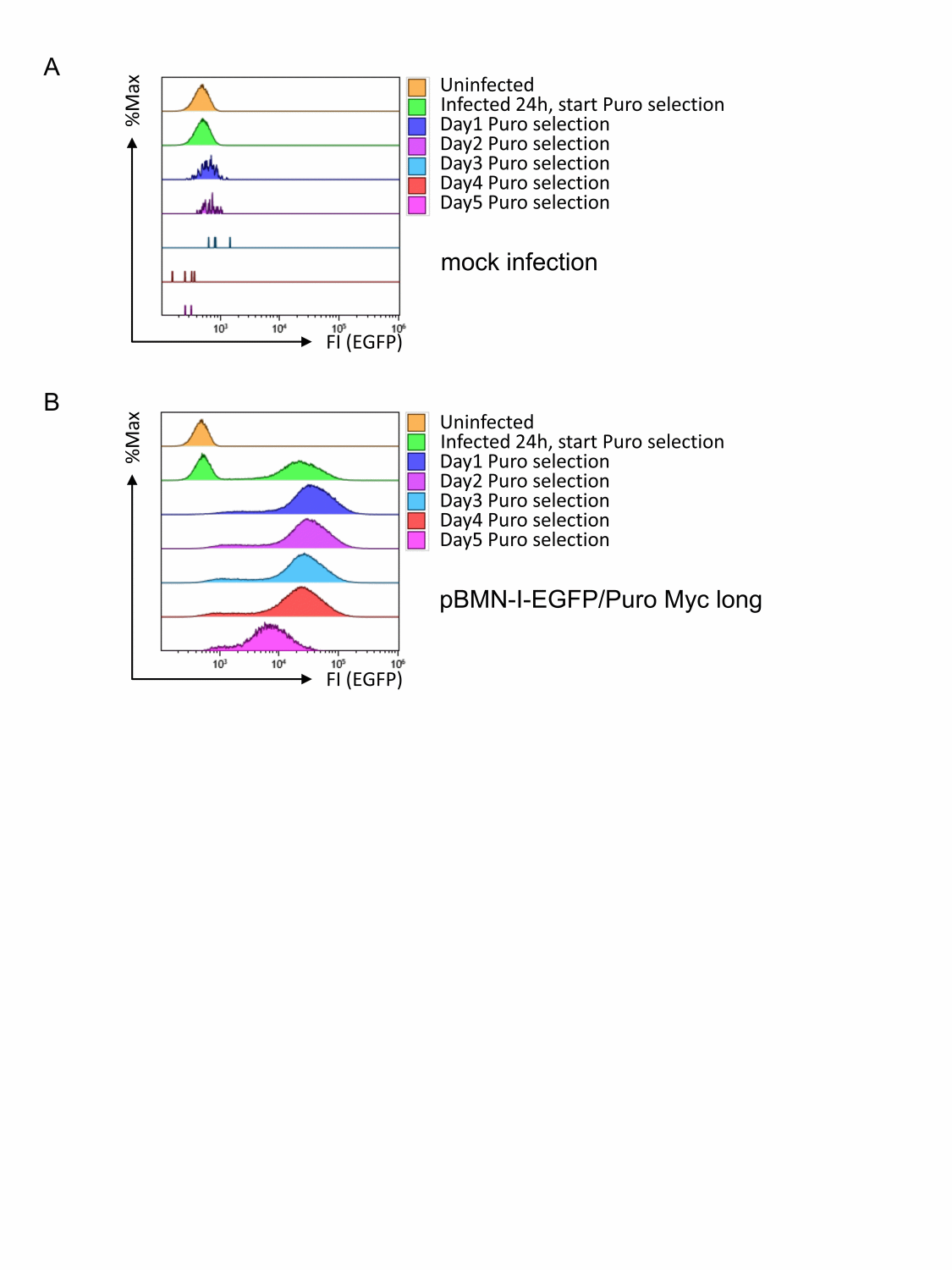

## Slide 13
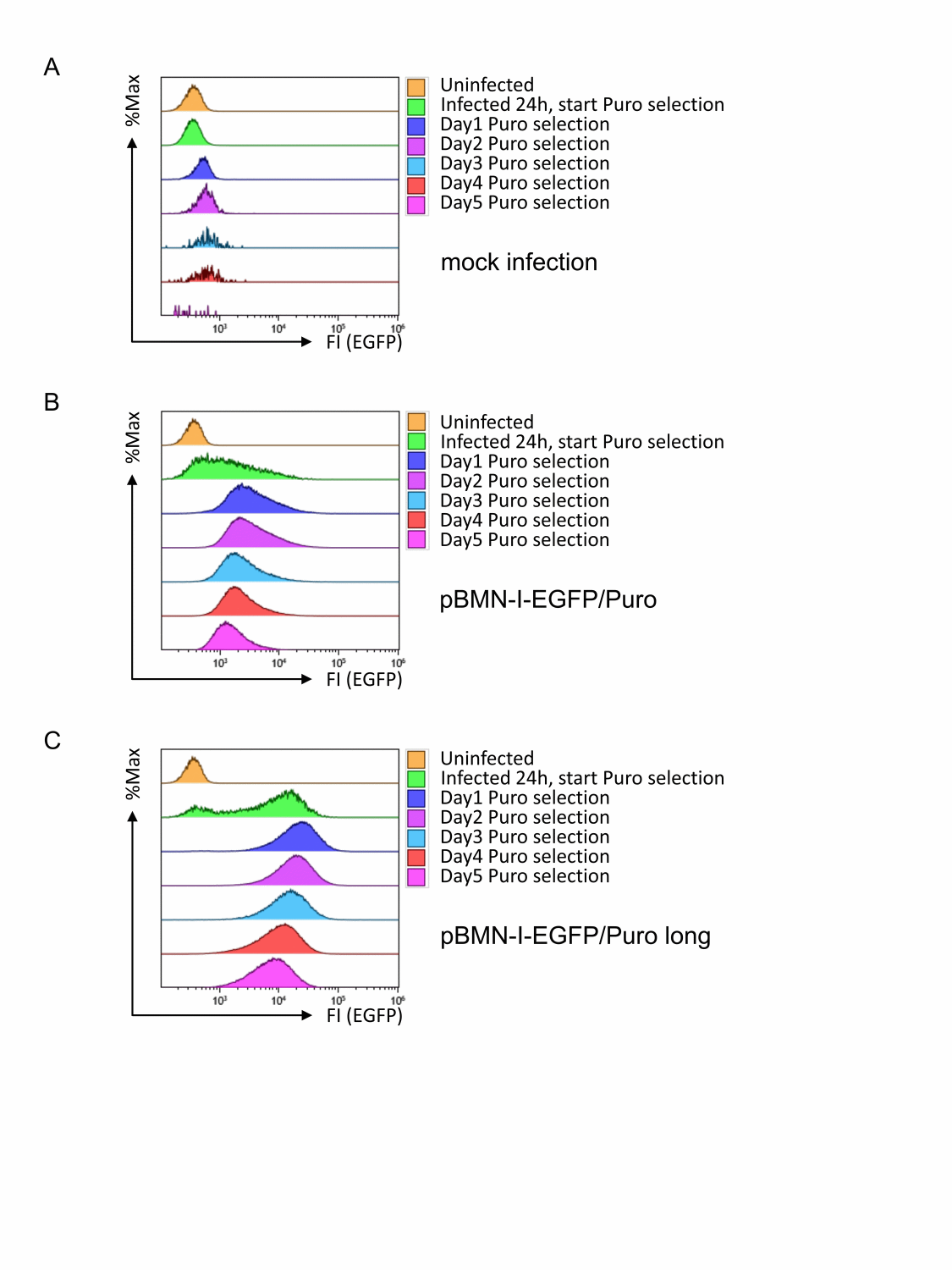

## Slide 14
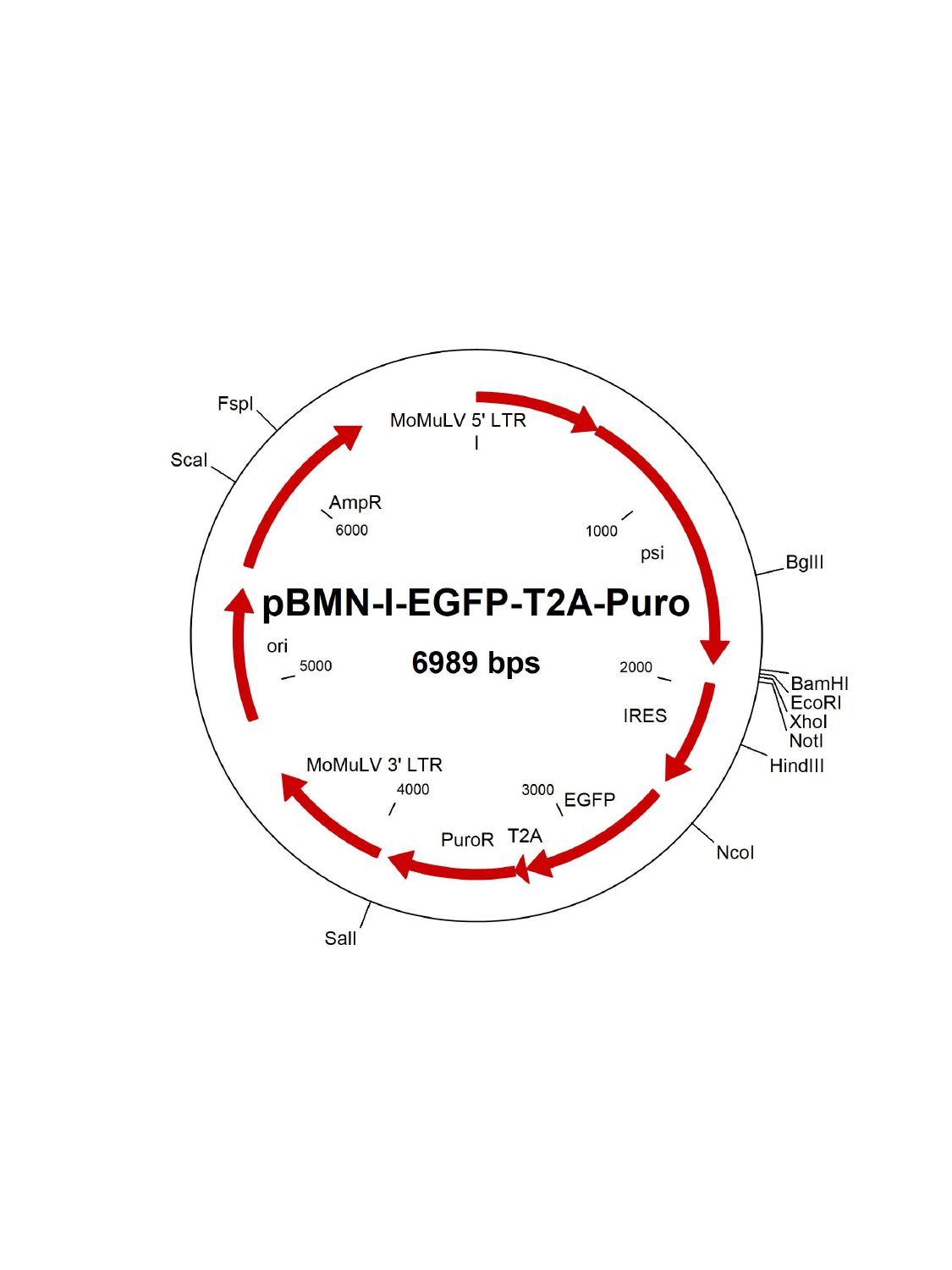

## Slide 15
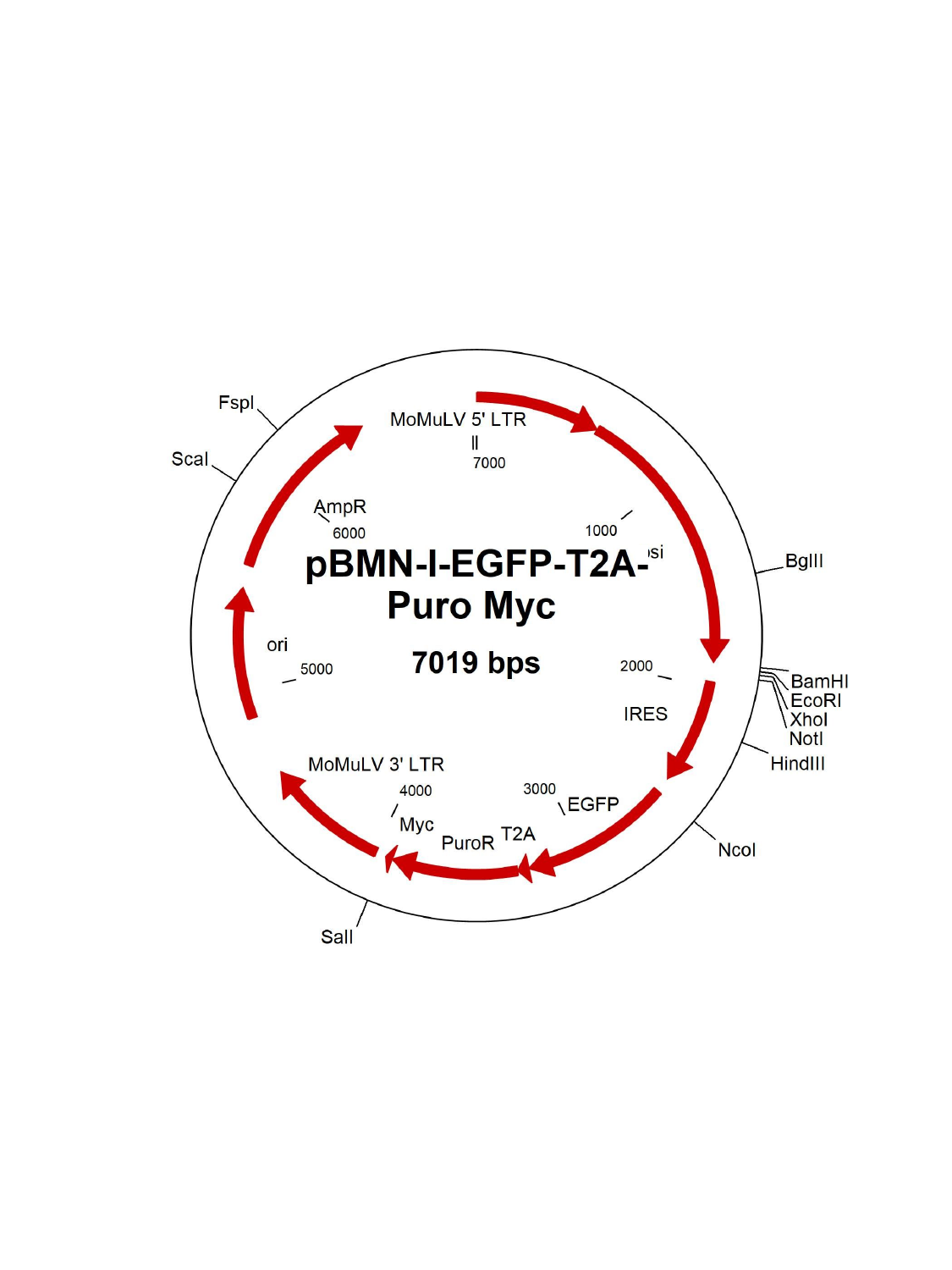

## Slide 16
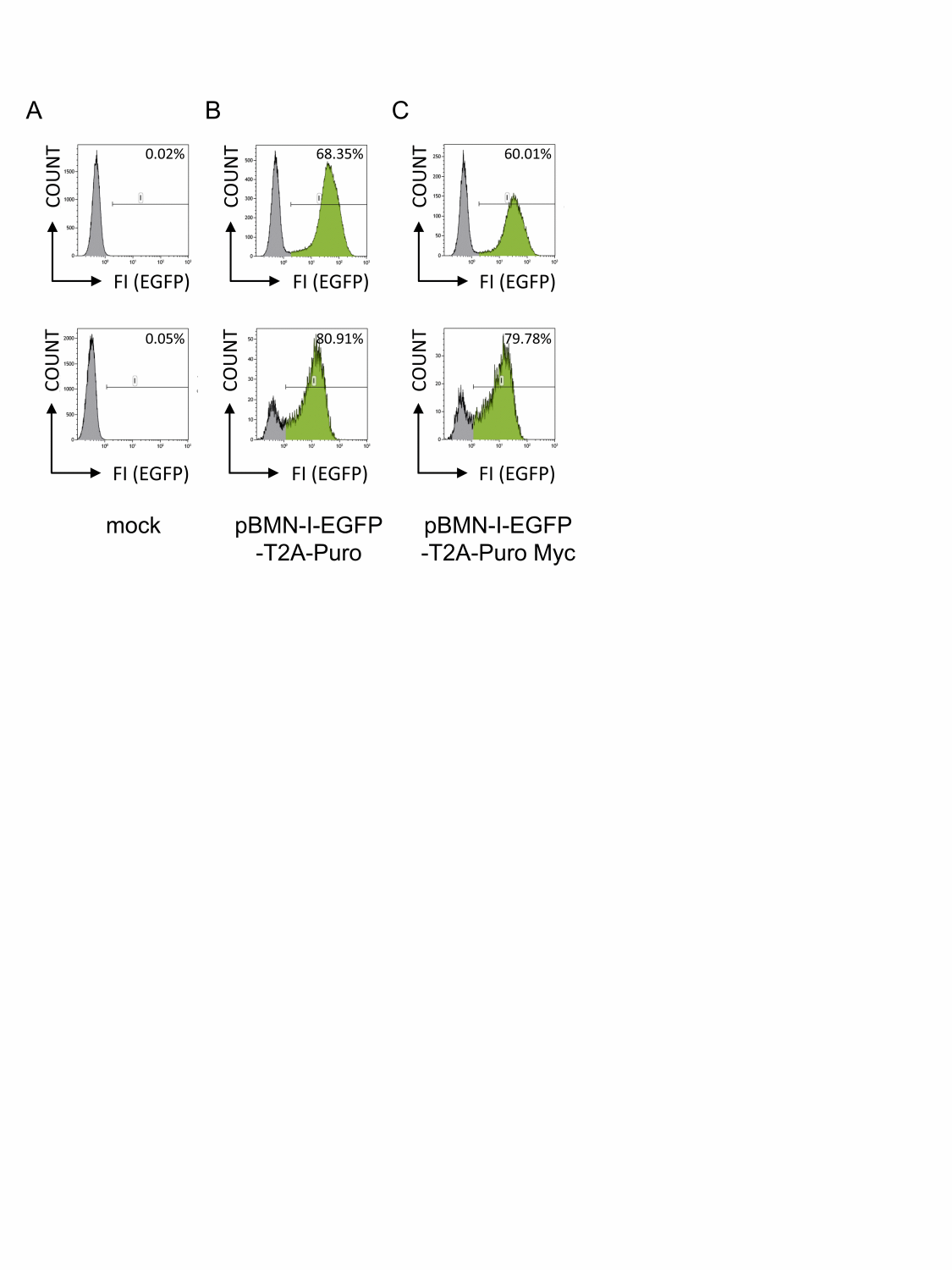

## Slide 17
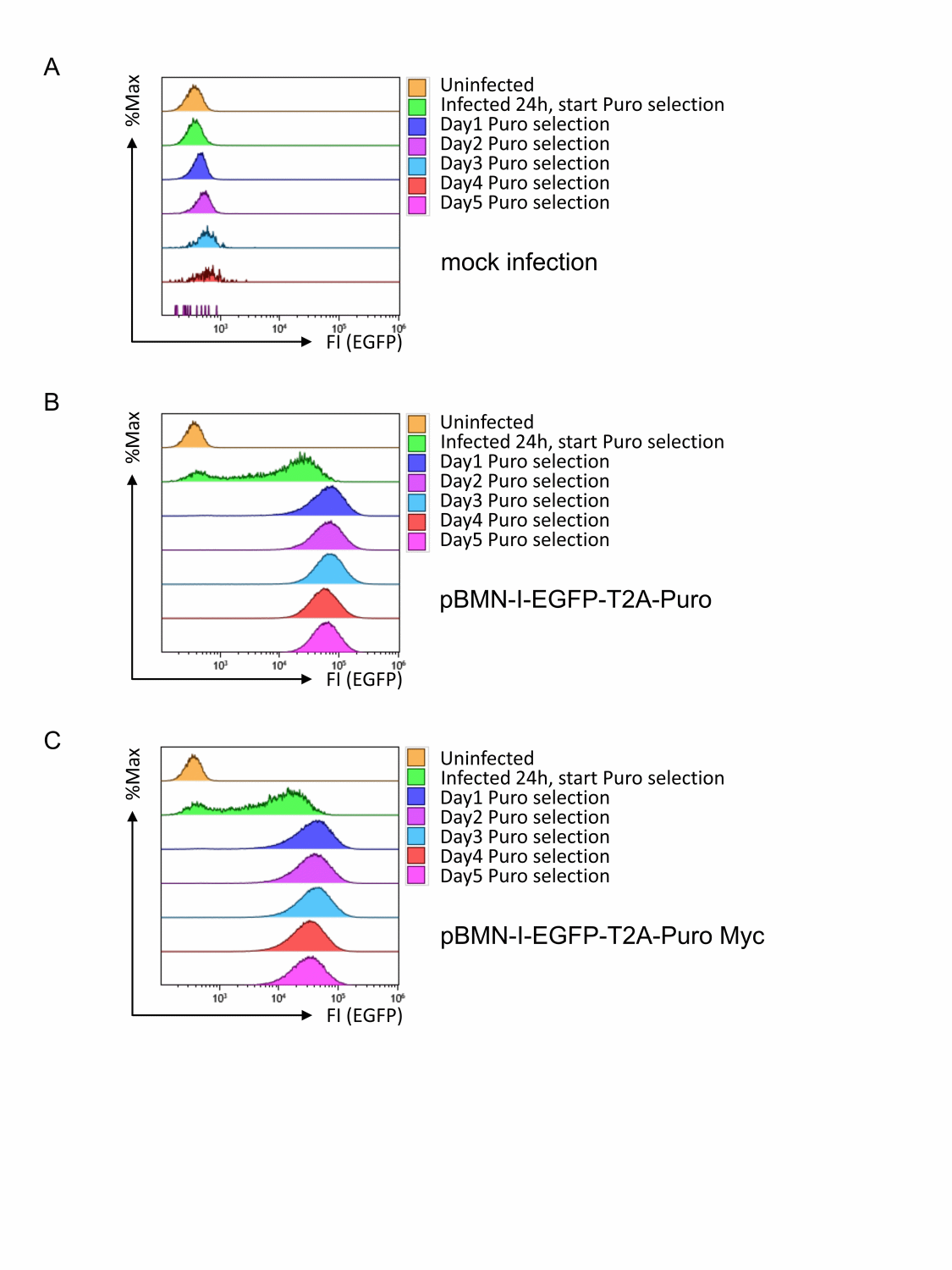

## Slide 18
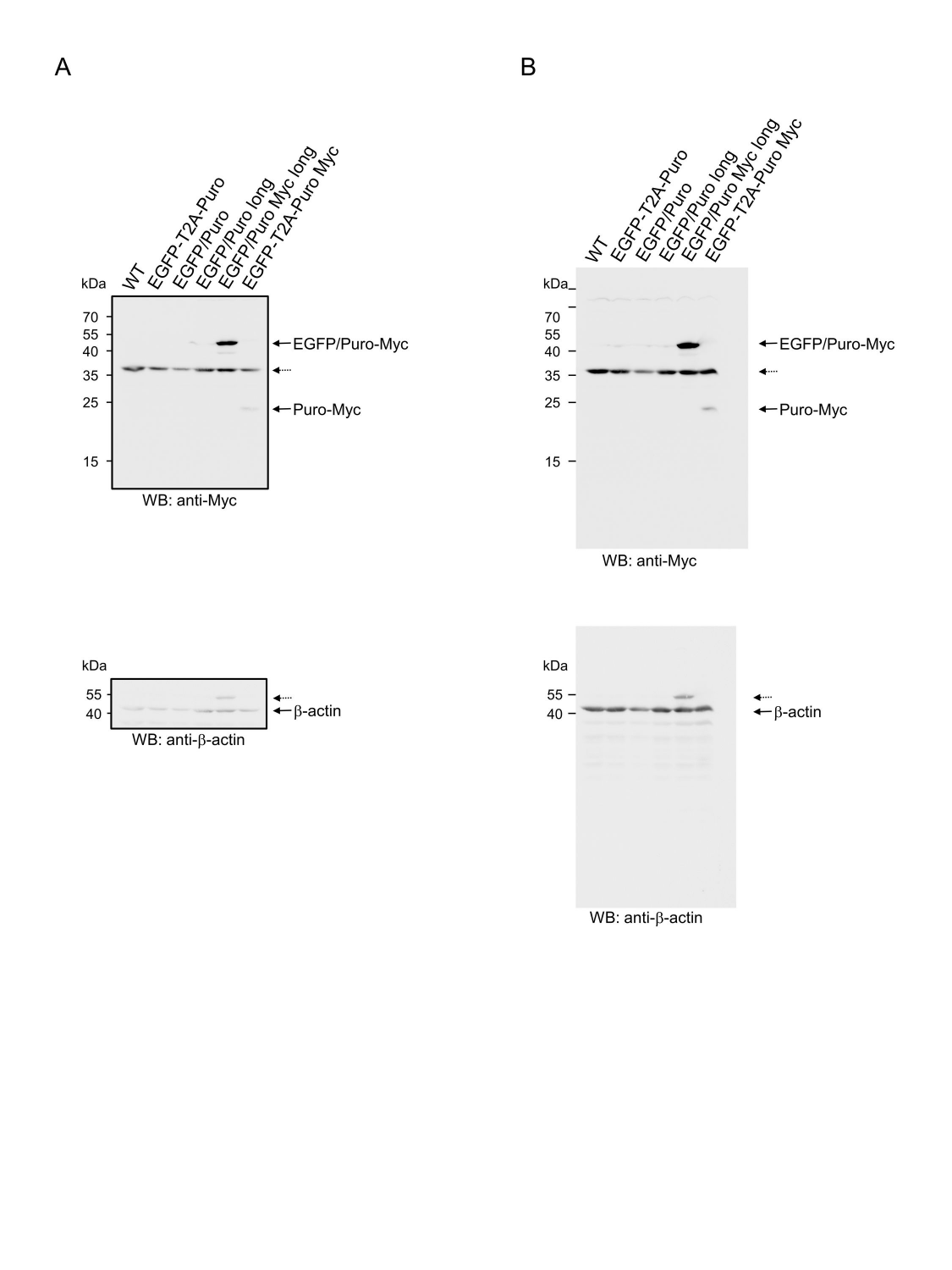

## Slide 19
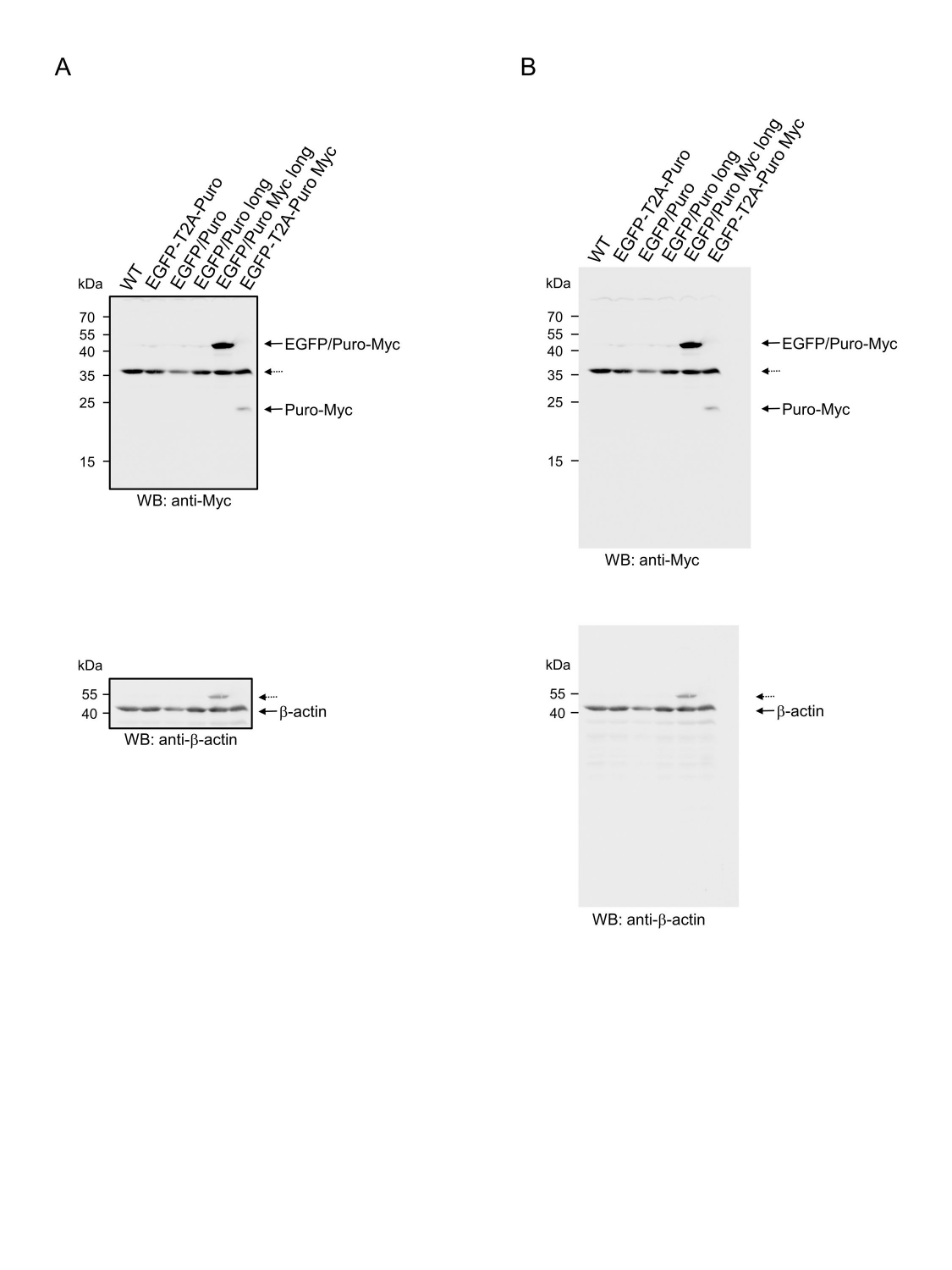

Supplement: Supplementary file 1 [file biomolecules-14-01131-s001.zip › Supplemental_Figures_Triller_et_al.pptx]
